# Transcriptome Remodeling of *Acinetobacter baumannii* during Infection and Treatment

**DOI:** 10.1128/mBio.02193-16

**Published:** 2017-03-07

**Authors:** Meredith S. Wright, Michael R. Jacobs, Robert A. Bonomo, Mark D. Adams

**Affiliations:** a The J. Craig Venter Institute, La Jolla, California, USA; b Department of Pathology, University Hospitals Cleveland Medical Center, Cleveland, Ohio, USA; c Department of Pathology, Case Western Reserve University, Cleveland, Ohio, USA; d Departments of Pharmacology, Molecular Biology, and Microbiology and Center for Proteomics, Case Western Reserve University, Cleveland, Ohio, USA; e Louis Stokes Cleveland Department of Veterans Affairs Medical Center, Cleveland, Ohio, USA; Northern Arizona University

## Abstract

*Acinetobacter baumannii* is an increasingly common multidrug-resistant pathogen in health care settings. Although the genetic basis of antibiotic resistance mechanisms has been extensively studied, much less is known about how genetic variation contributes to other aspects of successful infections. Genetic changes that occur during host infection and treatment have the potential to remodel gene expression patterns related to resistance and pathogenesis. Longitudinal sets of multidrug-resistant *A. baumannii* isolates from eight patients were analyzed by RNA sequencing (RNA-seq) to identify differentially expressed genes and link them to genetic changes contributing to transcriptional variation at both within-patient and population levels. The number of differentially expressed genes among isolates from the same patient ranged from 26 (patient 588) to 145 (patient 475). Multiple patients had isolates with differential gene expression patterns related to mutations in the *pmrAB* and *adeRS* two-component regulatory system genes, as well as significant differences in genes related to antibiotic resistance, iron acquisition, amino acid metabolism, and surface-associated proteins. Population level analysis revealed 39 genetic regions with clade-specific differentially expressed genes, for which 19, 8, and 3 of these could be explained by insertion sequence mobilization, recombination-driven sequence variation, and intergenic mutations, respectively. Multiple types of mutations that arise during infection can significantly remodel the expression of genes that are known to be important in pathogenesis.

## IMPORTANCE

Health care-associated multidrug-resistant *Acinetobacter baumannii* can cause persistent infections in patients, but bacterial cells must overcome host defenses and antibiotic therapies to do so. Genetic variation arises during host infection, and new mutations are often enriched in genes encoding transcriptional regulators, iron acquisition systems, and surface-associated structures. In this study, genetic variation was shown to result in transcriptome remodeling at the level of individual patients and across phylogenetic groups. Differentially expressed genes include those related to capsule modification, iron acquisition, type I pili, and antibiotic resistance. Population level transcriptional variation reflects genome dynamics over longer evolutionary time periods, and convergent transcriptional changes support the adaptive significance of these regions. Transcriptional changes can be attributed to multiple types of genomic change, but insertion sequence mobilization had a predominant effect. The transcriptional effects of mutations that arise during infection highlight the rapid adaptation of *A. baumannii* during host exposure.

## INTRODUCTION

Pathogens must overcome multiple selective pressures during host colonization and infection. Bacteria must survive not just antibiotic treatments but also host defenses, including nutrient limitation and host immune attack (1). Previous studies have demonstrated that pathogens mutate during persistent host infection, and many of these mutations are likely pathoadaptive (2–10). By using high-resolution genomic analysis of longitudinal series of *Acinetobacter baumannii* isolates, we recently found that newly arising nonsynonymous nucleotide substitutions and insertion sequence events were enriched in certain functional classes of genes, including two-component regulatory systems (TCRS) and other transcriptional regulators, iron acquisition and other transporters, and genes coding for surface-associated proteins like capsule and pilus genes (11). Here we explore how these mutations impact the transcriptome and whether there is evidence for convergence toward a common transcriptional profile in clinical *A. baumannii* strains. Population level genomic analysis of *A. baumannii* strains also reveals a dynamic genome modified by mutations, IS events, deletions, and gene acquisitions (12, 13). Therefore, we also assessed how population level genetic variation alters the transcriptome across major lineages. Understanding the impact of these genetic changes can reveal potential new targets for intervention that would impair the ability of *A. baumannii* to persist in the human host.

*A. baumannii* infections are increasingly difficult to treat because of high levels of antibiotic resistance in a majority of strains causing health care-associated infections (14). Previous studies have emphasized the importance of iron acquisition, transporters, and biofilm formation for virulence (15–17), but studies of human clinical isolates are limited. Transcriptional studies involving *A. baumannii* responses to iron or zinc limitation, antibiotic exposure, planktonic or biofilm conditions, and growth phase differences highlighted transcriptional changes in genes coding for type 1 pili (*csu* genes), biofilm formation, quorum sensing, and iron acquisition (18–21). Most of these studies used the ATCC 17978 strain as a model. ATCC 17978 has an unusual gene regulatory system mediated by plasmid-encoded transcriptional regulators where plasmid dynamics regulate chromosomal genes (22). Therefore, the transcriptional responses of clinical isolates with well-defined genetic variation remain largely unexplored.

In this study, we integrated high-resolution genomic variation with analysis of gene expression across single-isolate and population levels to examine how mutations that arise during infection and those that are maintained across distinct phylogenetic lineages can contribute to the transcriptional variation upon which selection can act.

## RESULTS

**FIG 1:**
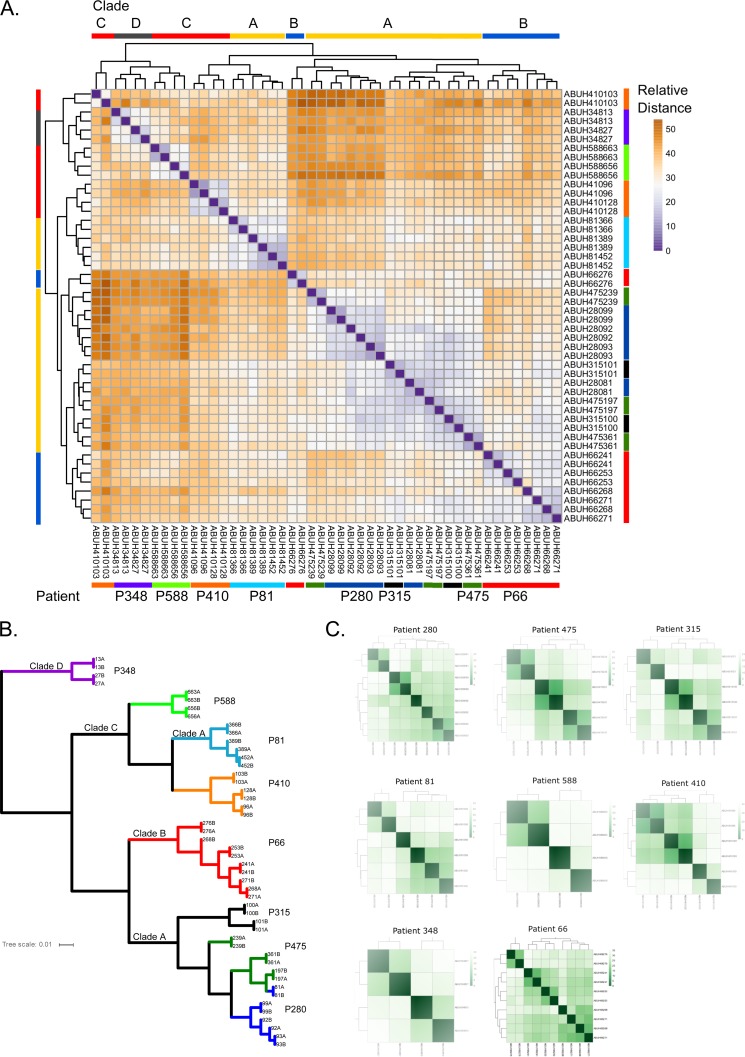
(A) Hierarchical clustering of VST count data from core clusters (*n* = 3,167 genes). (B) Hierarchical clustering of Spearman rank-based clustering from core clusters. (C) Clustering of VST data for all within-patient shared genes for each patient.

Differentially expressed genes were identified by using the defined thresholds in 24 *A. baumannii* isolates from eight patients and using comparisons among isolates from each patient (intrapatient differences) and among phylogenetic clades (interclade differences). Considering all eight intrapatient isolate sets, a total of 424 genes were differentially expressed in at least one patient and 127 of those genes were differentially expressed in more than one patient series. The number of differentially expressed genes ranged from 26 (patient 588, two isolates over 26 days) to 145 (patient 475, three isolates over 334 days) per patient (Table 1). In general, the expression patterns of isolates within each patient set were more like each other than like those of isolates from other patients on the basis of hierarchical clustering of the core gene transcriptome distance matrix, with a few notable exceptions (e.g., ABUH475239, ABUH410103, and ABUH66276) that are discussed below (Fig. 1A). Rank-based hierarchical clustering also indicated a strong phylogenetic signal in the transcriptional data, where each clade exhibited distinct sets of gene expression patterns (Fig. 1B). One exception was isolates from patient 81, which clearly belong to clade A in the core single-nucleotide variant (SNV) phylogeny but have several gene content differences from other clade A isolates (11). In some cases, a single isolate contributed most of the differentially expressed genes within a patient, for example, ABUH66726 in patient 66 and ABUH475239 in patient 475, whereas in the other patient sets, the variation in gene expression was distributed more evenly across all of the strains (Fig. 1C).

**TABLE 1:** Summary of isolate metadata, numbers of detected genetic changes, and numbers of genes differentially expressed within patients

Patient	Isolate	Strain	Date	Hospital	Ward	Source	Clade	No. of SNVs		No. of ISs		No. of differentially expressed genes	RNA-Seq SRA accession no.
								Isolate specific	Patient restricted	Isolate specific	Patient restricted		
66	241	ABUH66241	6/24/2008	Main	General	Miscellaneous	B	3	4	0		131	SRX1485325, SRX1485336
66	253	ABUH66253	7/16/2008	Main	General	Fluid	B	8		0			SRX1485338, SRX1485339
66	268	ABUH66268	8/13/2008	Main	General	Blood	B	0		0			SRX1485343, SRX1485341
66	271	ABUH66271	8/15/2008	Main	General	Wound	B	0		0			SRX1485356, SRX1485365
66	276	ABUH66276	8/24/2008	Main	ICU^a^	Fluid	B	12		3			SRX1485383, SRX1485385
81	366	ABUH81366	4/22/2009	Main	ICU	Sputum	A	3	5	0		41	SRX1562109, SRX1562113
81	389	ABUH81389	8/1/2009	Main	ICU	Sputum	A	2		0	1		SRX1562119, SRX1562120
81	452	ABUH81452	11/26/2009	Main	ICU	Sputum	A	1		0			SRX1562121, SRX1562122
280	81	ABUH28081	11/21/2007	Community A	General	Bronch	A	1	8	0		75	SRX1485405, SRX1485407
280	92	ABUH28092	12/4/2007	Community A	ICU	Bronch	A	0		0			SRX1485408, SRX1485410
280	93	ABUH28093	12/5/2007	Community A	ICU	Sputum	A	1		0			SRX1485413, SRX1485416
280	99	ABUH28099	12/20/2007	LTCF^b^	General	Sputum	A	2		0			SRX1485418, SRX1485419
315	100	ABUH315100	12/21/2007	Main	ICU	Sputum	A	2	0	0		33	SRX1485293, SRX1485291
315	101	ABUH315101	12/22/2007	Main	ICU	Bronch	A	2		0			SRX1485314, SRX1485304
348	13	ABUH34813	9/30/2007	Main	ICU	Sputum	D	4	0	2		66	SRX1562097, SRX1562100
348	27	ABUH34827	10/6/2007	Main	ICU	Sputum	D	3		3			SRX1562102, SRX1562107
410	96	ABUH41096	12/15/2007	LTCF	General	Sputum	C	0	2	0		124	SRX1562075, SRX1562081
410	103	ABUH410103	12/23/2007	LTCF	General	Sputum	C	0		3			SRX1562083, SRX1562082
410	128	ABUH410128	1/23/2008	LTCF	General	Trachea	C	0		2			SRX1562089, SRX1562084
475	197	ABUH475197	5/9/2008	Outpatient	Outpatient	Bronchus	A	0	0	1		145	SRX1485319, SRX1485318
475	239	ABUH475239	6/12/2008	Outpatient	Outpatient	Bronchus	A	1		2			SRX1485322, SRX1485324
475	361	ABUH475361	4/8/2009	Main	ICU	Stool	A	2		2			SRX1485401, SRX1485403
588	656	ABUH588656	7/4/2013	Main	ICU	Sputum	C	4	0	2		26	SRX1562123, SRX1562124
588	663	ABUH588663	7/30/2013	Main	ICU	Sputum	C	3		3			SRX1562126, SRX1562127

^a^ ICU, intensive care unit.

^b^ LTCF, long-term care facility.

Detailed below are examples of transcriptional variations among isolates from the same patient where a candidate within-patient genetic change could be hypothesized to lead to transcriptional variation of adjacent genes among isolates from the same patient, illustrating how genetic variation that arises during infection contributes to transcriptional remodeling among very closely related isolates. Several common themes were observed, with mutations in independent patients leading to transcriptional variation in a shared set of genes, suggesting convergent evolution of functional variation. This is followed by information on genetic mechanisms that underlie interpatient and population level transcriptional variations across more evolutionarily divergent isolates.

### Within-patient expression.

Putative genetic changes associated with transcriptional variation were found in the following patients.

### Patient 66.

**FIG 2:**
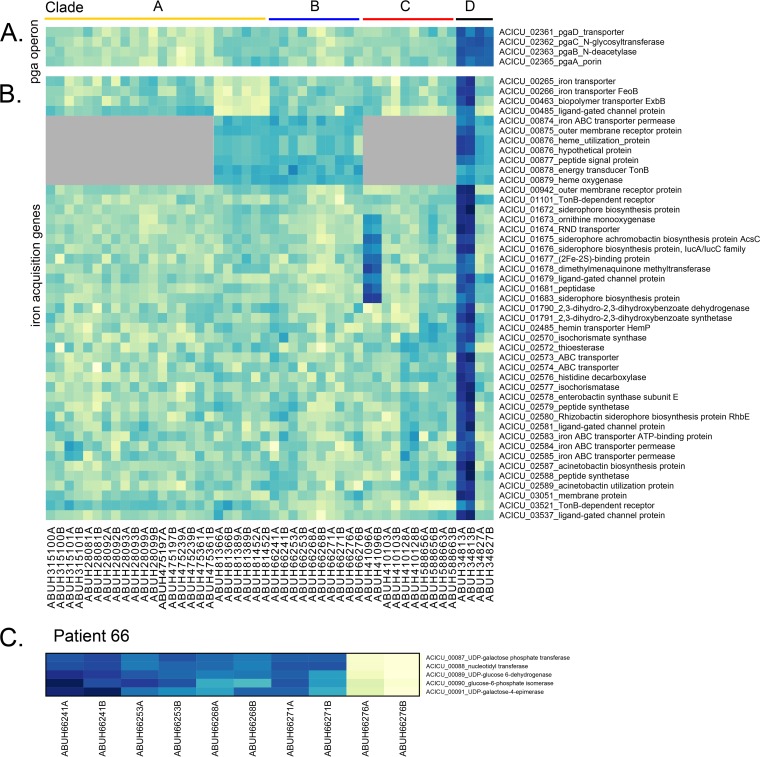
Expression (DESeq2-normalized counts) of differentially expressed genes in virulence regions. (A) Patient 348 isolates have an IS*Aba*1 insertion in common with other clade D isolates at the beginning of the *pga* operon. (B) ABUH34813 has a nonsynonymous mutation in the iron transporter gene *feoB* (ACICU_00266), and several genes related to iron acquisition were differentially expressed. A gene duplication of the siderophore region (ACICU_01673 to ACICU_01683) in ABUH41096 led to significantly higher expression of those genes. (C) An IS*Aba*125 insertion upstream from ACICU_00087 in ABUH66276 led to significantly lower expression of cps synthesis genes. Color intensity is scaled within each panel, with darker shades indicating higher expression; see Table S7 for values. Gray shading indicates genes absent from those isolates.

Five strains isolated over 61 days had 27 SNVs and three IS events among them, and 132 differentially expressed genes were identified (Table 1). Isolate ABUH66276, the last isolate in the series, accounted for most of the differentially expressed genes (see Table S1 in the supplemental material), and many of these genes were adjacent to IS and mutation events (see Tables S2 and S3). An IS*Aba*125 insertion upstream from ACICU_00087 likely resulted in decreased transcription of ACICU_00087 to ACICU_00091, genes involved in capsular polysaccharide (cps) biosynthesis (Fig. 2C). An IS*Aba*1 insertion in ACICU_01765, a transcriptional regulator, was associated with significantly decreased expression of ACICU_01762, a predicted aspartate ammonia lyase, in ABUH66276. ABUH66276 also had IS*Aba*1 inserted upstream from ACICU_01814, which likely causes the overexpression of ACICU_01812 and ACICU_01813, involved in pilus assembly, a region also disrupted by patient-restricted IS*Aba*1 in patient 41, upstream of ACICU_01813. IS*Aba*1 has a strong outward-facing promoter that can result in overexpression of the upstream flanking gene when oriented in the opposite direction. A predicted EamA-like transporter, ACICU_03323, is also more highly transcribed in ABUH66276, though sequences within patient 66 isolates are identical across 5 kb of sequence flanking the gene. ABUH66276 has two other transporters with patient-specific SNVs (ACICU_03495 and ACICU_01674) and an IS*Aba*1 insertion in a gene encoding another membrane protein (ACICU_03345). ABUH66276 has a nonsynonymous mutation in ACICU_03477, a predicted 4-aminobuytrate transaminase, and the expression of adjacent ACICU_03476 to ACICU_03479, part of the 4-aminobutyrate catabolic pathway, is significantly increased. IS-associated deletion of ACICU_01775 to ACICU_01779 was present in ABUH66276, flanked downstream by an IS*Aba*1 insertion in ACICU_01780 common to all clade C isolates and upstream by an additional isolate-specific IS*Aba*1 insertion at ACICU_01774, in a region encoding a transcriptional regulator, transporters, and dehydrogenases of unannotated specificity. Point mutations in ACICU_03495 and ACICU_03498 (a TCRS hybrid sensor histidine kinase/response regulator) in ABUH66276 are associated with decreased transcription of genes in this cluster, including a predicted membrane transport system. ACICU_01781 (predicted to be part of a phenol degradation pathway) is more highly expressed in patient 66 isolates than in the other isolates likely because of the IS*Aba*1 insertion in ACICU_01780 (a flavin reductase gene).

10.1128/mBio.02193-16.1 TABLE S1  Differentially expressed genes within each patient. The values reported are mean DESeq2-normalized mapped read counts. Download TABLE S1, PDF file, 0.3 MB. Copyright © 2017 Wright et al. 2017 Wright et al. This content is distributed under the terms of the Creative Commons Attribution 4.0 International license.

10.1128/mBio.02193-16.2 TABLE S2  SNVs identified in each isolate and patient. Download TABLE S2, PDF file, 0.05 MB. Copyright © 2017 Wright et al. 2017 Wright et al. This content is distributed under the terms of the Creative Commons Attribution 4.0 International license.

10.1128/mBio.02193-16.3 TABLE S3  Insertion sequence mapping summary for each isolate. Insertion sequence locations were determined with ISseeker (12). Download TABLE S3, PDF file, 0.01 MB. Copyright © 2017 Wright et al. 2017 Wright et al. This content is distributed under the terms of the Creative Commons Attribution 4.0 International license.

TCRS mutations were also present in isolates from this patient: *pmrB* (ABUH66253) and two independent *adeS* mutations (ABUH66253 and ABUH66276) discussed in further detail below.

### Patient 81.

**FIG 3:**
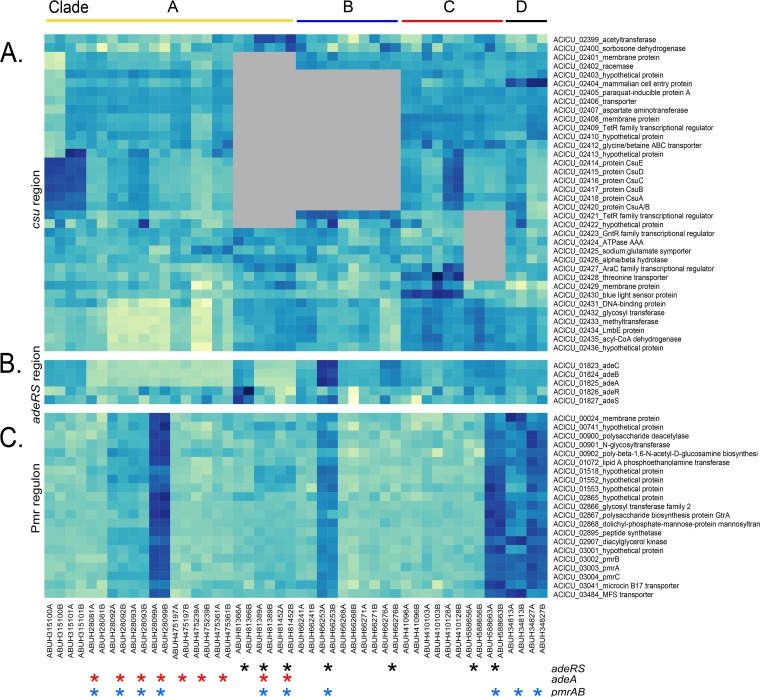
Gene expression (DESeq2-normalized counts) in regions of convergent change. (A) *csu* (type I pilus secretion) and flanking regions. See the text for details of genetic changes. (B, C) *adeRS* and *adeABC* region (B) and Pmr regulon (C): differentially expressed genes common to *pmrAB* mutants. Color intensity is scaled within each panel, with darker shades indicating higher expression; see Table S7 for values. Asterisks denote isolates with mutations. Gray shading indicates genes absent from those isolates.

In patient 81, three isolates differed by six SNVs and one insertion sequence event that occurred during the 218 days of patient infection, with 41 differentially expressed genes among them (Table 1; see Table S1). ABUH81366 had mutations in two efflux systems, *adeR* (encoding the response regulator) of the AdeABC efflux pump and early termination of *adeJ* of the AdeIJK RND family efflux pump, while the other two isolates from this patient had a 5-bp deletion upstream of *adeA* (ACICU_01825) (see Table S2). The *adeABC* genes have ~25-fold lower expression in ABUH81389 and ABUH81452 than in other isolates with the wild-type genotype (Fig. 3B), while ABUH81366 did not differ in transcription across this region. The predicted early termination of *adeJ* in ABUH81366 likely led to the significantly lower expression of the downstream gene *adeK*. A *pmrB* mutation (T114I) is present in ABUH81389 and ABUH81452, and the *pmrCAB* operon has ~4× higher expression in these strains than in ABUH81366 (Fig. 3C). An insertion sequence-associated mutation in ACICU_03611 (see Table S3) was associated with increased expression of the ACICU_03609-to-ACICU_03614 genes in ABUH81389 and ABUH81452, a region encoding predicted amino acid metabolism. Plasmid-carried genes were also more highly transcribed in ABUH81389 and ABUH81452.

### Patient 280.

Four isolates spanning 29 days had 74 differentially expressed genes among them, with 12 SNVs and no IS events occurring during this time (Table 1). The first isolate, ABUH28081, was the most divergent, but its transcriptional profile was more like that of the closely related patient 475 isolates (Fig. 1B). Each isolate had mutations in *pmrB*, with a gradient of transcriptional effects, with ABUH28099 having the greatest increase in *pmrCAB* gene expression (Fig. 3C; see Tables S1 and S2). ABUH28081 had an isolate-specific *pmrB* SNV, while the other three isolates shared a different *pmrB* mutation and ABUH28099 had an additional isolate-specific SNV in *pmrB*.

### Patient 315.

Two isolates obtained 1 day apart had 33 differentially expressed genes and four SNVs between them (Table 1) and gene content differences, including partial loss of the Tn*1548*-like transposon encoding the aminoglycoside resistance gene *armA* in ABUH315100. Increased expression of ACICU_01471 to ACICU_01475 (ubiquinol oxidase genes) was observed in ABUH315101, with a mutation in the adjacent gene encoding a LysR family transcriptional regulator (ACICU_01470). Phage-like genes in ABUH315101 were also more highly transcribed. Other genetic variation between the two isolates included a mutation in ACICU_03157, encoding a histidine kinase transcriptional regulator, a gene also mutated in ABUH475239, but there was no observed transcriptional variation in flanking genes in either isolate.

### Patient 348.

We identified 66 differentially expressed genes between two isolates obtained 6 days apart from patient 348 (Table 1). Both isolates have an SNV in *pmrB* in common with all clade D isolates and showed elevated transcription of Pmr regulon genes (Fig. 3C). ABUH34813 has an additional mutation in a predicted lipopolysaccharide export permease (ACICU_00253), while ABUH34827 has a nonsynonymous mutation in a predicted lipid A export permease (ACICU_01602) (see Table S2), which may explain the lower expression across the Pmr regulon genes (Fig. 3C) relative to ABUH34827. ABUH34813 had an additional mutation in the ferrous iron transporter gene *feoB* (ACICU_00266), and the transcription of iron acquisition genes, including multiple siderophore genes, was significantly elevated in this isolate (Fig. 2B). ABUH34813 had an IS*Aba*125 interruption of ACICU_00745, where transcription of the adjacent ACICU_00746 and ACICU_00747 genes was significantly lower. Isolate ABUH34827 had an IS*Aba*12-associated deletion in the *csu* region (ACICU_02420 and the 5′ end of ACICU_02421) (see Table S3), and transcript levels across this region (ACICU_02414 to ACICU_02421) were significantly lower (Fig. 3A). Both isolates have an IS*Aba*12 insertion within ACICU_01721, *rnd*, encoding RNase D (also IS*Aba*125 in ABUH475239). ABUH34827 has an additional IS*Aba*12 insertion in ACICU_01726 that likely led to the deletion of ACICU_01721 to ACICU_01726, encoding hypothetical proteins and a transcriptional regulator, in ABUH34827.

### Patient 410.

Three isolates spanning 39 days had 123 differentially expressed genes among them, with the intermediate isolate (ABUH410103, day 8) having the more divergent transcriptome than the other two isolates (Table 1; Fig. 1C). Duplication of a siderophore gene region in ABUH41096 likely resulted in significantly higher expression of these genes (Fig. 2B). Genomic read mapping reveals >2× coverage of ACICU_01673 to ACICU_01685 (340× for ABUH41096 versus 130× for the other two patient 410 genomes across this region). There was a frameshift (1-bp deletion) in a gene encoding a component of the shikimate pathway (ACICU_03387) in the ABUH410103 genome, which also had significantly elevated transcription of the phenylacetic acid (*paa*) pathway (ACICU_01135 to ACICU_01345). These transcriptional changes may represent a response to aromatic amino acid limitation. An intergenic IS*Aba*1 between ACICU_01103 and ACICU_01104 in ABUH410103 (see Table S3) was associated with lower expression of the adjacent genes, a predicted dihydroorotase (ACICU_01103) and RNase T (ACICU_01102). ABUH410103 also had an IS*Aba*125 insertion within ACICU_02538, encoding an RNA methyltransferase. ABUH410128 had an IS*Aba*1 upstream of ACICU_02420, *csuA*, likely leading to higher expression of the chaperone usher system genes (Fig. 3A). The mutations in RNA methyltransferase and RNase T may, in part, contribute to the divergent transcriptome in ABUH410103.

### Patient 475.

Three isolates collected over 334 days had 145 differentially expressed genes among them, with three SNVs and five insertion sequence mobilization events distinguishing them (Table 1). ABUH475239 (day 34) had the most divergent transcriptome (Fig. 1C). An IS*Aba*125 insertion near the C terminus of RNase D (*rnd*, ACICU_01720) at amino acid (aa) 360 was identified in this isolate (see Table S3), and expression of the adjacent gene’s hypothetical protein, ACICU_01721, was lower. An IS*Aba*125 insertion in ACICU_02613, a LysR transcriptional regulator, had no apparent effect on the adjacent predicted choline transporters, but the expression of ACICU_00888 to ACICU_0891 was significantly elevated. This region also contains genes predicted to be involved in choline transport and betaine biosynthesis. ABUH475239 also had an intergenic IS*Aba*125 insertion between ACICU_00288 and ACICU_00289, and the transcription of the adjacent gene, ACICU_00289, a putative DNA binding protein upstream from a type II secretion system region, was significantly higher. There is also an example of intergenic sequence length variation likely associated with transcriptional variation, where ABUH475239 is missing 76 bp between ACICU_02427 (AraC-type DNA binding domain protein) and ACICU_02428 (predicted threonine or homoserine lactone transporter), adjacent to *blsA*, encoding a blue light sensor domain (BLUF) protein (ACICU_02430) (23). Many genes were differentially expressed in ABUH475239 relative to the other two isolates (Fig. 3A), but no single mutations could be identified as likely causes of these differences. It is possible that the RNase D mutation altered tRNA processing or that a potential phase change around the ACICU_02427 region is linked to the expression differences.

### Patient 588.

Two isolates obtained 26 days apart had 26 differentially expressed genes and were distinguished by seven SNVs and five IS locations (Table 1). The two *pmrB* mutations in ABUH588663 (see Table S2) likely contributed to many of the differentially expressed genes. ABUH588663 also had a nonsynonymous mutation in a predicted metal transporter (ACICU_00170). This isolate had 6× greater expression of ACICU_03142, encoding a TonB receptor domain protein, although no mutations were identified in the flanking region that might explain the expression difference. ABUH588565 had an IS*Aba*27 insertion in *carO*, ACICU_02813 (see Table S3), encoding an outer membrane protein, and the expression of that gene was significantly lower. CarO disruption, including by insertion sequences, has been shown to result in increased carbapenem resistance (24–27).

### Population level genetic and transcriptomic variations.

To further explore how genome-wide genetic changes can alter expression at the population level, the most variably expressed genes were identified by using two statistical models (i) where each isolate was independent and (ii) where isolates were assigned to a clade on the basis of the core SNV phylogeny in reference 11. Clade level differences in gene expression were clustered in 42 colocated blocks comprising 68 differentially expressed genes. Of those 42 blocks, a rationale for the causative mutation could be inferred on the basis of genetic differences in *cis* for 30 of the loci: 19 with insertion sequence events, 8 with nonsynonymous coding changes, and 3 with intergenic changes. Of the 130 most variable genes across all of the isolates, there were 78 blocks of gene clusters with consistent transcriptional patterns. Of these blocks, 21 were linked to insertion sequence events, 11 were linked to sequence divergence, and 9 were linked to single-base nonsynonymous mutations. There were 32 genes identified as both highly variable across all of the isolates and having clade-differentiated expression levels.

### Convergent changes. (i) *pmrAB* mutations: defining the Pmr regulon in *A. baumannii*.

Nine isolates from five patients had mutations in genes encoding TCRS PmrAB that encode a known mechanism conferring resistance to colistin in *A. baumannii* through lipid A modification. All four isolates from patient 280 had *pmrB* mutations: ABUH28081 had an isolate-specific SNV, ABUH28092 and ABUH28093 shared a second *pmrB* mutation, and ABUH28099 shared this site and had an additional mutation (see Table S2). Differential expression along this gradient indicated that the V31F mutation conferred an ~5× higher level of transcription of *pmrC*, the gene encoding lipid A phosphoethanolamine transferase, in ABUH28092 and ABUH28093, while the additional L257I mutation in ABUH28099 resulted in 30× higher expression of *pmrC* (Fig. 3C). All of the isolates had the same colistin MIC of 0.5 µg/ml, as tested in a broth microdilution assay. Transcriptome analysis indicated that isolates carrying *pmr* mutations had 21 differentially expressed genes in common that help define the Pmr regulon in *A. baumannii* (Fig. 3C). These genes have considerable overlap with the colistin-regulated genes described by Cheah et al. (28). Interestingly, none of the isolates with *pmr* mutations had *in vitro* colistin MICs of >1 µg/ml and were classified as phenotypically susceptible.

### (ii) *adeRS* mutation: contrasting transcriptional patterns.

Mutations in the TCRS genes *adeRS* and in the *adeABC* efflux pump genes, which are associated with tigecycline resistance (29–32), were associated with both elevated and reduced expression (Fig. 3B). Clade A isolates from both patients 280 and 475 had an IS*Aba*22 insertion in *adeA* (ACICU_01825) that likely rendered it nonfunctional, and expression of the *adeABC* operon was significantly lower. Clade A isolates from patient 81 also had mutations in this region, including a patient-specific mutation in *adeS* (ACICU_01827). There was an isolate-specific mutation in ABUH81366 in *adeR* (A80P), while the two later isolates (ABUH81389 and ABUH81452) have a 5-base deletion −40 bp upstream from *adeA*, with *adeABC* expression significantly higher in ABUH81366 than in the other isolates from patient 81. Phenotypically, the tigecycline MIC for ABUH81366 was 16 µg/ml, while the MIC for both ABUH81389 and ABUH8155 was 4 µg/ml. A patient-specific *adeR* mutation in patient 588 was associated with ~2× greater transcription of *adeABC*. Patient 66 had two isolates with independent *adeS* mutations, with ABUH66253 (D167N) having ~6× greater *adeABC* expression than ABUH66276 (R312S), yet the transcription of this region was significantly higher in both of these isolates than in the other three isolates from patient 66. The tigecycline MIC for ABUH66253 was 16 µg/ml, compared to 8 µg/ml for ABUH66276. No additional gene expression effects of *adeRS* mutations were apparent.

### (iii) Type I pilus alteration: *csu* region.

The genome region with the greatest variability in gene expression was a 37-kb region containing the *csu* genes (ACICU_02399 to ACICU_02436) (see Table S7). The Csu proteins make up a type I pilus chaperone usher secretion system that is important for biofilm formation and adhesion (33, 34). The *csu* region was interrupted by deletions, IS events, and SNVs (see Tables S2 and S3). The comparison of isolates within clade C from patients 588 and 410 highlights this. An IS*Aba*27 interruption of *csuB* (ACICU_02417) is unique to patient 588. There is an isolate-specific IS*Aba*1 insertion between ACICU_2420 and ACICU_2421 in ABUH410128 associated with increased expression of ACICU_02414 to ACICU_02421 relative to that in the other two isolates from patient 410 (Fig. 3A). Isolates from both patients have IS interruptions within ACICU_02430 (blue light sensor domain protein). The ACICU_02430 insertions involve unique locations in this gene and involve different IS elements (IS*Aba*125 in patient 410 and IS*Aba*27 in patient 588). Isolates from both patients have an IS*Aba*1 insertion between ACICU_02428 and ACICU_02429 in common, but the upstream flanking sequence adjacent to the IS*Aba*1 element, including ACICU_02421, is deleted from the patient 588 strains. The transcription of this region reflects the influence of these elements, as isolates from patients 410 and 588 have elevated transcription relative to the other isolates from ACICU_02430 (patient 410 isolates) or ACICU_02431 (patient 588 isolates) through ACICU_02436 (Fig. 3A). This region contains additional genes that may be involved in the structure of the cell surface, including a predicted glycosyltransferase and a gene with a conserved domain associated with glycosyl-phosphatidylinositol anchor biosynthesis.

In clade D patient 348, there is an isolate-specific IS*Aba*12 insertion within ACICU_02421, a transcriptional regulator, in ABUH34827, with decreased expression of the *csu* operon in this isolate relative to that in ABUH34813. Patient 66 (clade B) is missing ACICU_02401 to ACICU_02421, while patient 81 (clade A) is missing ACICU_ 02398 to ACICU_ 02423, because of IS*26*-mediated deletions. ABUH315100 (clade A) has an isolate-specific IS*26*-associated deletion starting at ACICU_02398 (major facilitator superfamily transporter) through ACICU_02414 (*csuE* gene). Patient 315 also has a patient-specific mutation in *csuE* with two amino acid differences from other clade A isolates. Genomic and transcriptomic analyses reveal a dynamic remodeling of this region with a consistent trend toward loss or diminished function of the *csu* region.

### (iv) Type 1 pilus alteration, take 2: ACICU_01812 to ACICU_01815*.*

Additional convergent mutations occurred in another set of genes encoding components of the type I pilus system. All of the isolates in clades A to C have an IS*Aba*1 insertion (at aa 6) within ACICU_01812, a pilus assembly protein. An isolate-specific mutation in ACICU_01814 in ABUH66276 is associated with the overexpression of adjacent ACICU_01813, also a pilus assembly protein. Isolates from clade D have an intergenic deletion of 417 bp between ACICU_01813 and ACICU_01814. Since all of the isolates have mutations relative to ACICU, understanding what wild-type expression of this region looks like is not possible, but these genes differ significantly from each other in expression. Other isolates from the UHHS population have IS*Aba*1 insertions in this region as well: isolate-specific IS*Aba*1 in ABUH35559 and patient-restricted IS*Aba*1 in isolates from patient 41.

### (v) Convergent mutation and IS-mediated alterations of ACICU_01919 to ACICU_01923*.*

Clade C isolates from patient 588 have two IS insertions in the ACICU_01919-to-ACICU_01923 region: IS*Aba*27 in ACICU_01919 (a predicted amidase) and IS*Aba*1 in ACICU_1921 (transcriptional regulator). ACICU_01919 and ACICU_01920 transcription is significantly lower in patient 588 isolates, but ACICU_01921 transcription is increased. However, the insertions are within the coding region, so ACICU_01919 and ACICU_01920 are not likely functional in these isolates. Clade B isolates from patient 66 have a 1-bp deletion in ACICU_01920, a predicted quinol monooxygenase, that causes a frameshift at aa 56, but transcriptional effects of this are equivocal. The clade D isolates from patient 348 isolates are missing over 70 kb of sequence (ACICU_01890 to ACICU_01950), including this region. The deletion was possibly due to a recombination between IS*Aba*1 elements because one edge of the deleted region is marked by an IS*Aba*1 insertion in ACICU_01890, annotated as hemolysin D.

### IS-mediated mobilization of *osmC* and *crp* genes.

A subset of strains from clades A to C have an IS*Aba*1 insertion near the end of ACICU_01161, predicted to be *crp*, encoding the cyclic AMP receptor protein, a transcription factor. Clade A strains have an additional IS*Aba*1 downstream of ACICU_01162, an *osmC*-like gene predicted to be involved in the osmotic stress response. A composite transposon composed of IS*Aba*1, *crp*, *osmC*, and IS*Aba*1 appears to be readily mobilized and was found in several strains in distinct locations. Clade A strains have a second copy in ACICU_01570 (hypothetical periplasmic protein), and a third copy was found in ACICU_03149 (a hypothetical protein in the outer core locus for lipooligosaccharide synthesis) of the clade A patient 280 and 475 strains. In the latter strains, the expression of ACICU_01161 and ACICU_01162 was significantly higher, while clade D strains without duplications of these genes had the lowest expression (see Table S6). The PacBio assembly of ABUH28099 (AYOH01000000) (13) contains three copies of this transposon and confirms this mobilization.

### Other IS effects at the patient and clade levels.

Patient 348 isolates share a clade D-specific IS*Aba*1 near the beginning of *pgaA* (ACICU_02365 at aa 39), encoding the poly-β-1,6 *N*-acetyl-d-glucosamine export protein, which is important for biofilm formation (35), and significantly increased transcription of the adjacent ACICU_02361-to-ACICU_02365 genes relative to the other isolates (Fig. 2A; see Table S7). Patient 410 has patient-specific IS*Aba*1 in ACICU_01189 at aa 206 (out of 261 aa), and the expression of both that gene and the adjacent gene for ACICU_01188, annotated as a benzoate transporter, is increased. An example of convergence in insertion sequence events affects *rnd*, encoding RNase D (ACICU_01720): an IS*Aba*12 insertion is present in all clade D isolates, and an isolate-specific IS*Aba*125 event was found near the C terminus in clade A isolate ABUH475239.

### Intergenic mutations.

Intergenic mutations can change expression by altering promoter or DNA binding sites without changing gene function. A mutation 4 bp upstream of the start codon for ACICU_00684 (a predicted membrane protein) in clade C isolates, is associated with significantly higher expression of this gene, though there is unexplained variation in the expression of this gene among the clade A isolates as well. Of additional note, the ACICU genome has IS*Aba*125 inserted upstream of this gene. Clade B isolates all have a mutation −40 bp upstream from the start codon of ACICU_00708, encoding a diguanylate cyclase domain protein, and expression of this gene is significantly higher in clade B strains than in the others.

### Sequence variation through recombination.

Clade level variation in expression of genes not explained by insertion sequence or single-base differences can also be attributed to sequence variation or regions of elevated sequence divergence indicative of historical recombination events (see Table S5). Because the strains investigated here are all part of multilocus sequence type 2 (ST2) or the global clone II lineage, the average nucleotide identity in core genes is often >99.9%. However, some genomic regions have elevated SNV density (13, 15, 36). One of these regions starts around 3.51 Mbp (36) and encompasses several genes identified as differentially expressed at the clade level: ACICU_03220 to ACICU_03627 (see Table S5), encoding proteins involved in pilus assembly, amino acid metabolism, and environmental sensing and response proteins. For example, in ACICU_03431 and ACICU_03432, clade D isolates are identical to ACICU, missing 11 aa within a membrane protein (ACICU_03432), where clade A to C sequences are identical to TYTH-1 (M3Q_3669). Transcription is significantly lower in the clade D isolates. A 314-bp region is variably present among *A. baumannii* genomes where these extra bases start at ACICU coordinate 3650446 in the transmembrane protein ACICU_03437 of clade A to C isolates (as well as clade E isolates in reference 13) and identical to the AC29 finished genome. This 314-bp sequence is absent from clade D isolates, which are identical to ACICU here. Clade B isolates also have an IS*Aba*1 insertion that interrupts ACICU_03437. Transcription of this region is significantly higher in patient 348 clade D isolates, while the flanking Eam-like transporter domain ACICU_03436 and cold shock protein domain ACICU_03438 are also variably expressed. A third location of expression variance in this region is around ACICU_03471. Isolates in clades A to C are identical across this 2,538-bp region, while clade D isolates are instead all 2,534 bp long, with frameshifts present in ACICU_03471, a predicted *S*-adenosylmethionine-dependent methyltransferase. Sequence conservation is low here, however, where clades A to C have 36 single-nucleotide polymorphisms (SNPs) over this ~2.5-kb region relative to ACICU as well. An isolate-specific IS*Aba*125 insertion is also present in ABUH410103 in ACICU_03472 (a NAD-flavin adenine dinucleotide binding domain protein) and is associated with decreased expression of ACICU_03471.

10.1128/mBio.02193-16.4 TABLE S4  Genes identified as differentially expressed among clades. The putative mutation and type of mutation associated with variability are listed. Blocks of genes with the same expression patterns are shaded in gray. The values reported are mean DESeq2-normalized mapped read counts. Download TABLE S4, PDF file, 0.03 MB. Copyright © 2017 Wright et al. 2017 Wright et al. This content is distributed under the terms of the Creative Commons Attribution 4.0 International license.

10.1128/mBio.02193-16.5 TABLE S5  The most variably expressed core genes among isolates. The putative mutation and the type of mutation associated with the variability are listed. Blocks of genes with the same expression pattern are shaded in gray. Values reported are mean DESeq2-normalized mapped read counts. Download TABLE S5, PDF file, 0.04 MB. Copyright © 2017 Wright et al. 2017 Wright et al. This content is distributed under the terms of the Creative Commons Attribution 4.0 International license.

## DISCUSSION

Bacteria face selective pressures from host defenses and antibiotic therapies during colonization and infection. We previously identified mutations that arose during infection, finding enrichment in certain classes of genes, including transcriptional regulators, transporters, and iron acquisition- and surface-associated structures (11). In this study, we examined how mutations can contribute to transcriptional variation within and among patients and across phylogenetically distinct lineages. Transcriptional changes among isolates could be linked to a combination of single base substitutions (intergenic and intragenic), insertion sequence mobilization, IS-associated deletions, duplications, and recombination events. We found that closely related isolates can have very different transcriptional profiles and that transcriptional changes can arise over the time frame of patient infection. Additionally, there is convergence at the transcriptome level, with independent genetic changes causing shared patterns of transcriptional remodeling of regions associated with antibiotic resistance, iron acquisition, biofilm formation, surface structures like pili and cps architecture, and amino acid metabolism. The clade level comparison identified expression differences that reflect evolutionary divergence, and many of these differences can be attributed to insertion sequence events and recombination-associated sequence variation in the same genes with altered expression in the within-patient series.

Genetic analysis indicated an enrichment in TCRS mutations during host infection (11). Regulatory networks are complex, and mutations in one system can have effects through cross-talk among regulatory networks (37–40). Multiple independent occurrences of *pmrAB* mutations allowed us to more rigorously establish the Pmr regulon in clinical *A. baumannii* isolates, which identified 18 additional genes whose transcription was altered in the mutant strains, including several hypothetical proteins and sugar transferases, including those that may be involved in galactosamine modification of lipid A (41). Most isolates with *pmr* mutations originated from patients treated with colistin (11), supporting the inference that these mutations arose in response to colistin selective pressure. However, the *pmrB* mutation common to all clade D isolates represents a lineage-specific variation that may confer reduced susceptibility to colistin.

A second TCRS mutated in multiple patients is *adeRS*. The contrasting transcriptional patterns of mutations around the *adeRS* and *adeABC* loci highlight the potential adaptive trade-off surrounding antibiotic resistance and fitness. Yoon et al. (42) quantified the fitness cost of increased production of *A. baumannii* efflux pumps, as a strain with increased expression of *adeABC* demonstrated less *in vitro* and *in vivo* fitness than the wild-type strain. Previous genomic surveys also indicated substantial genetic variation in *adeRS* (30), which is not always associated with a tigecycline resistance phenotype, highlighting that this region is likely experiencing multiple sources of selection. A recent study examined the transcriptional response of *A. baumannii* in *adeRS* deletion strains (20). Several other operons were found to be differentially expressed, a pleiotropic effect not observed in this study, though this may be because deletion mutants were investigated in that study. Feugeas et al. (43) demonstrated that genetic diversity correlates with transcriptional variation under *in vitro* growth conditions for *Escherichia coli* cells, including an enrichment in TCRS, illustrating the significant impact mutations these genes can have on transcription and fitness.

Multiple independent genetic events in the same region are evidence of the potential adaptive significance of these regions. For example, the *csu* region has been altered multiple independent times in *A. baumannii* (13, 15) and we observed both increased and decreased expression relative to that in isogenic isolates from several patients. In an analysis of IS locations in over 1,000 *A. baumannii* genomes, the 5-kbp region containing the *csu* genes had over 90 independent insertion events, a density approximately three times higher than that in any other 5-kb region of the genome (12). This provides compelling evidence that alteration of this region is under selection. The *csu* region is known to be critical for biofilm formation and adhesion (34) and therefore thought to be important for virulence, but the prevalence of deletions and transcriptional downregulation also suggests that under certain conditions, expression of the Csu proteins might be selected against or that this region may be under diversifying pressure. A second type I pilus system also experienced convergent genetic and transcriptional changes. Mutations in type 1 fimbriae have been described in *Shigella* spp. that led to loss of function, and yet fimbrial production increased epithelial cell invasion success (44), suggesting a complex interplay of selective pressures acting on these loci.

Iron acquisition is also critical for within-host *A. baumannii* survival (45, 46). Two isolates had mutations in iron acquisition: ABUH41096 (duplication across the siderophore synthesis region) and ABUH34813 (point mutation in the ferrous iron transporter FeoB). Both isolates had significantly increased expression of the siderophore region, ACICU_01672 to ACICU_01683, while ABUH41096 had increased expression across an additional 36 iron acquisition-related genes (Fig. 2B), suggesting iron limitation in this isolate even during growth in rich laboratory medium. Free ferrous iron is likely limited during host infection (47), so the *in vivo* fitness implications of this mutation remains to be tested, though the two subsequent isolates (ABUH410103 and ABUH410128) from the patient did not have this mutation.

Though convergence was observed among patients, within-patient analysis indicated that transcription in most isolates varied only in a limited number of genes. One exception is ABUH66276 from patient 66, which had many more differentially expressed genes (Fig. 1C). This isolate also had the most isolate-specific mutations: 12 SNVs and three insertion sequence events. A potential explanation for this divergence of expression patterns involves the cumulative effects of *recA*, *rpoB*, and *rne* RNase (E/G) mutations that likely impact the transcription of DNA damage-inducible proteins and RNA processing (48). This example highlights that an isolate can experience rapid transcriptome remodeling through a relatively small number of genetic changes.

Many of the gene expression differences observed in isogenic sets of isolates from individual patients could be associated with specific mutations in adjacent genomic regions. A larger number of expression differences affected genes without obvious candidate mutations. There are at least two explanations for this. First, regulatory networks are complex and pathway interactions are incompletely characterized in *A. baumannii*, especially for nonmodel strains. We also lacked the power to detect *trans*-acting mutations. Second, although biological replicate data sets were highly similar for each isolate (Fig. 1C; see Table S7), subtle growth phase effects could have influenced transcriptional profiles. For example, 11 of the most variable genes among isolates involved the phenylacetic acid (*paa*) pathway, a pathway whose expression is known to be tightly coupled to the growth phase (49). Furthermore, transcriptional effects of some mutations were likely not detected under the *in vitro* growth conditions used here, where additional expression differences may be detected *in vivo*. Finally, expression data do not directly equate to whether a gene has lost its function or changed a phenotype, and additional phenotypic characterization of these isolates would yield important information regarding the pathoadaptive significance of these mutations. For example, clade D isolates share genetic and transcriptional alterations of the biofilm-associated *pga* region and the Pmr regulon such that the surface properties of these cells are likely to be very different from those of other *A. baumannii* strains.

10.1128/mBio.02193-16.6 TABLE S6  DESeq2-normalized counts for the composite transposon region including IS*Aba1*, *crp*, *osmC*, and IS*Aba1*. Download TABLE S6, PDF file, 0.02 MB. Copyright © 2017 Wright et al. 2017 Wright et al. This content is distributed under the terms of the Creative Commons Attribution 4.0 International license.

10.1128/mBio.02193-16.7 TABLE S7  Raw read counts mapped to ACICU (CP000863.1) genes. Download TABLE S7, XLSX file, 0.9 MB. Copyright © 2017 Wright et al. 2017 Wright et al. This content is distributed under the terms of the Creative Commons Attribution 4.0 International license.

This analysis highlights how genetic changes can significantly alter the transcription of phenotypically relevant genes, including those associated with antibiotic resistance, iron acquisition, and surface-associated properties, among highly similar isolates. That multiple mechanisms of genetic change contributed to this variation underscores the importance of analyzing all types of genetic variation, not only SNVs, to understand how bacterial genomes reflect the selective pressures that cells experience.

## MATERIALS AND METHODS

### Isolates and patient data.

This research study was reviewed and approved by the Institutional Review Board of the University Hospitals Case Medical Center and conformed to the Helsinki Declaration. A subset of isogenic isolates from the longitudinal series from the University Hospitals Health System (UHHS) in Ohio, described in reference 11, was selected for RNA sequencing (RNA-Seq) to represent a range and type of mutational events, as well as to have at least one representative series from each primary clade in references 11 and 13 (Table 1). All of the isolates belong to ST2. These 24 isolates originated from eight patients, and duplicates of each from single colonies were grown overnight in LB broth, transferred to fresh medium, and harvested at mid-log growth phase (optical density at 600 nm of 0.5). Cells were then pelleted (2 min at 13,000 × *g*), the supernatant was removed, and the pellet was flash frozen in liquid nitrogen. Cell pellets were stored at −80°C before RNA was extracted with the MagJET RNA purification kit (Thermo Fisher) with an additional DNase treatment. rRNA was depleted with RiboZero (Illumina). Paired-end RNA-Seq libraries were made with PrepX kits on the Wafergen Apollo liquid handling system and sequenced on the Illumina NextSeq platform.

### RNA-Seq methods.

RNA-Seq reads were mapped with CLC Genomic Workbench (Qiagen) to each isolate’s respective genome assembly and to the *A. baumannii* ACICU complete reference genome (CP000863.1) to minimize the effect of assembly differences in the draft genomes. Each isolate had >99.7% average nucleotide sequence identity to ACICU. By using the option to “Map to gene regions only,” each read pair (or RNA fragment) was counted as aligning to the single gene with the longest alignment. Orthologs among isolates were identified with PanOCT (50), and core genes among these isolates were operationally defined as those present in ACICU and at least 23 of the study isolates (*n* = 3,167 genes). Raw fragment counts for each gene were output from CLC Genomic Workbench for statistical analysis in DESeq2. Genes were identified as differentially expressed if their adjusted *P* value was <0.05 and the log_2_-fold change was greater than |1.5| (i.e., a >3-fold change) to minimize artifacts associated with multiple-comparison testing with statistical models comparing isolates within a patient or among phylogenetic clades. To additionally identify gene expression variability among individual isolates, the most variable core genes were defined as those that had >1 standard deviation from the mean variance-stabilizing transformed (VST) DESeq2 count data. This equates to 130 genes or ~4% of the core ACICU predicted coding regions.

### Genetic variation detection.

SNVs were identified as described in reference 11. Briefly, genomic reads were mapped to the first isolate from the series and SNVs were called with FreeBayes (arXiv:1207.3907v2). Insertion sequence events were identified with ISseeker (12). Clade level genetic differences were identified by assembly-based SNV calling in kSNP (51) and verified manually to confirm that variants were isolate, patient, or clade specific by using blast queries with all of the available *A. baumannii* genomes in the UHHS population (11, 13).

### Accession number(s).

The RNA-Seq reads and genome assemblies obtained in this study are publicly available from the NCBI under BioProject no. PRJNA262565.
