# Supplementary material for: Transcriptome Remodeling of Acinetobacter baumannii during Infection and Treatment
Source: mBio. 2017 Mar 7;8(2):e02193-16. doi: 10.1128/mBio.02193-16 (PMC5340874; doi:10.1128/mBio.02193-16)
Supplement: TABLE S1 [file mbo001173221st1.pdf]

Patient 66

Locus Tag	Predicted Protein	DE in # of other patients	ABUH66241	ABUH66253	ABUH66268	ABUH66271	ABUH66276
ACICU_00053	hypothetical protein		124.90	16.38	15.22	13.55	18.22
ACICU_00061	oxidoreductase	3	21.61	12.79	86.45	29.95	100.18
ACICU_00062	fatty acid desaturase	3	53.90	35.11	172.87	67.52	260.33
ACICU_00087	UDP-galactose phosphate transferase		599.95	518.15	592.10	603.25	45.66
ACICU_00088	nucleotidyl transferase		1007.92	763.28	953.22	1089.81	85.77
ACICU_00089	UDP-glucose 6-dehydrogenase		710.28	563.32	639.66	542.98	142.52
ACICU_00090	glucose-6-phosphate isomerase		545.36	488.88	384.56	449.36	170.03
ACICU_00091	UDP-galactose-4-epimerase		1694.82	1045.56	1362.47	1139.07	422.51
ACICU_00095	lactate dehydrogenase	3	779.97	333.90	1487.88	902.11	652.10
ACICU_00475	preprotein translocase		71.17	60.99	18.65	20.13	101.74
ACICU_00571	NAD(P) transhydrogenase subunit alpha	2	257.45	160.07	529.89	758.03	105.22
ACICU_00572	NAD(P) transhydrogenase subunit alpha		41.50	22.06	70.45	92.06	13.03
ACICU_00573	NAD synthetase		208.08	130.11	359.33	465.80	70.23
ACICU_00594	acyl-CoA synthetase	2	544.90	491.63	314.20	267.81	101.11
ACICU_00662	acyl-CoA dehydrogenase		339.55	316.64	493.92	432.20	142.62
ACICU_00678	5-methyltetrahydropteroyltriglutamate--	homocysteine me	648.84	256.18	982.30	748.90	1189.23
ACICU_00684	membrane protein	4	1023.92	638.35	783.57	484.22	84.41
ACICU_00761	trehalose-6-phosphate synthase	3	8057.01	3824.25	3247.87	3099.47	676.16
ACICU_00762	trehalose phosphatase	6	489.52	230.81	80.94	114.00	22.88
ACICU_00861	gamma-glutamyltransferase	2	506.26	551.04	705.04	383.21	711.93
ACICU_00888	choline dehydrogenase	3	786.24	795.77	2251.59	1160.01	4508.25
ACICU_00889	betaine-aldehyde dehydrogenase	2	1115.53	1297.04	2559.25	1651.95	3827.99
ACICU_00890	BetI family transcriptional regulator	2	971.97	1189.61	1987.74	1351.72	3107.91
ACICU_00891	choline transporter	2	369.61	495.09	691.38	396.99	1231.43
ACICU_00900	polysaccharide deacetylase	3	634.58	1788.40	674.95	639.62	468.12
ACICU_00901	N-glycosyltransferase	3	526.63	1158.79	482.76	472.90	364.49
ACICU_00958	esterase		56.55	72.43	19.36	55.29	9.05
ACICU_01027	hypothetical protein	4	336.20	132.09	58.14	132.47	45.12
ACICU_01085	amino acid transporter	3	95.19	58.00	35.97	35.40	123.42
ACICU_01192	benzene 1,2-dioxygenase		121.77	153.80	114.13	98.84	36.14
ACICU_01198	cysteine-rich helical bundle repeat protei	4	14151.94	5554.72	6599.69	7556.01	1658.15
ACICU_01214	hypothetical protein	3	503.07	418.19	242.08	391.85	106.46
ACICU_01215	hypothetical protein	3	343.87	187.23	86.52	244.30	40.72
ACICU_01216	head morphogenesis protein	3	503.35	250.18	207.56	282.44	73.44
ACICU_01221	hypothetical protein	3	906.88	405.53	572.92	574.08	76.19
ACICU_01261	type VI secretion system protein	3	826.82	812.51	922.42	942.66	595.77
ACICU_01266	membrane protein	2	1974.99	1995.32	896.64	1046.47	579.23
ACICU_01268	hypothetical protein	2	1774.31	1614.63	934.21	815.23	378.86
ACICU_01270	allophanate hydrolase	2	920.09	1066.53	542.80	544.84	336.05
ACICU_01272	voltage-gated chloride channel protein		75.94	66.70	36.68	54.94	169.29
ACICU_01407	2-isopropylmalate synthase		1481.78	1518.12	2278.80	1511.49	537.73
ACICU_01408	3-methylcrotonyl-CoA carboxylase		1810.34	1804.31	2703.85	1956.69	698.33
ACICU_01409	enoyl-CoA hydratase		908.78	959.87	1264.44	943.08	324.54
ACICU_01411	isovaleryl-CoA dehydrogenase		1413.37	1403.12	1958.59	1694.16	636.76
ACICU_01420	hypothetical protein	2	134.24	126.69	40.87	82.00	32.50
ACICU_01421	hypothetical protein	2	115.66	72.23	69.14	84.70	10.20
ACICU_01422	hypothetical protein	2	7661.59	3716.13	4732.37	5261.69	674.46
ACICU_01423	hypothetical protein	4	4854.43	2524.06	2805.23	3805.46	456.85
ACICU_01425	hypothetical protein	3	6290.15	2514.64	3039.90	4062.26	1104.87
ACICU_01426	hydroperoxidase	3	30036.13	15784.62	18685.37	20461.61	7275.68
ACICU_01427	short-chain dehydrogenase	3	746.24	330.76	332.58	434.04	125.23
ACICU_01505	Na+:H+ dicarboxylate symporter		786.35	651.74	485.03	316.56	48.59
ACICU_01517	hypothetical protein		33.65	94.36	20.80	37.90	36.03
ACICU_01518	hypothetical protein	5	140.09	4404.26	109.26	114.05	61.53
ACICU_01552	hypothetical protein	5	17.46	1679.61	4.15	10.80	12.80
ACICU_01553	hypothetical protein	4	7.66	115.84	9.69	13.01	13.01
ACICU_01706	porin		56.35	121.45	40.10	27.94	113.46
ACICU_01762	aspartate ammonia-lyase		6246.04	4212.85	4204.26	3459.83	21.73

ACICU_01767	acetyl-CoA acetyltransferase		1467.29	1152.77	2160.98	1482.19	520.64
ACICU_01769	succinyl-CoA:3-ketoacid-CoA transferase		2117.85	2077.46	3575.96	1840.08	865.81
ACICU_01770	succinyl-CoA:3-ketoacid-CoA transferase		487.83	503.36	578.65	377.27	162.66
ACICU_01801	hypothetical protein	3	97.63	49.98	35.96	54.26	13.26
ACICU_01805	hypothetical protein	4	78.44	36.49	25.58	24.06	9.52
ACICU_01813	pilus assembly protein fimA	2	384.03	467.03	540.36	392.38	1653.31
ACICU_01822	peptidase S15		19.43	111.50	20.75	24.50	49.05
ACICU_01823	adeC putative outer membrane efflux prc	2	112.26	2375.57	255.23	192.90	829.20
ACICU_01824	adeB cation/multidrug efflux pump	2	624.35	10328.23	903.91	842.12	2594.07
ACICU_01825	adeA membrane fusion protein	3	305.23	4901.96	382.56	340.48	943.85
ACICU_01911	hemagglutinin	3	249.72	353.21	442.61	356.29	1478.31
ACICU_01912	hemolysin activator protein	4	61.75	173.90	193.00	155.93	646.01
ACICU_01935	alpha/beta hydrolase	2	3150.59	3036.16	4082.84	4121.37	1179.62
ACICU_01975	acyl-CoA dehydrogenase	2	55.10	38.19	20.07	14.04	4.18
ACICU_01976	glutamyl-tRNA amidotransferase	3	263.63	145.18	94.71	64.82	34.69
ACICU_02071	hypothetical protein	4	135.09	67.73	56.68	80.82	22.30
ACICU_02270	hypothetical protein	3	81.73	86.88	43.56	59.75	17.08
ACICU_02276	hypothetical protein	4	164.43	87.72	76.84	122.94	19.72
ACICU_02289	hypothetical protein	2	317.38	232.41	69.83	82.78	94.96
ACICU_02347	AMP-binding protein		657.90	735.07	912.35	878.10	94.28
ACICU_02348	acyl-CoA dehydrogenase		282.75	278.06	436.20	341.72	44.73
ACICU_02349	3-hydroxy-2-methylbutyryl-CoA dehydrogenase		555.95	499.35	690.87	732.38	75.72
ACICU_02358	glycine zipper		343.66	94.88	86.43	77.18	71.09
ACICU_02385	hypothetical protein		139.98	93.71	71.92	133.54	29.80
ACICU_02431	DNA-binding protein	3	843.95	493.04	616.84	529.92	125.89
ACICU_02432	glycosyl transferase	3	523.60	211.22	285.57	390.64	82.63
ACICU_02433	methyltransferase	3	318.81	118.39	128.63	189.90	43.17
ACICU_02434	LmbE protein	3	542.27	190.24	301.41	338.83	55.58
ACICU_02435	acyl-CoA dehydrogenase	3	302.22	114.36	175.59	181.20	31.60
ACICU_02436	hypothetical protein	3	5120.29	5179.19	2331.85	4314.46	941.53
ACICU_02437	gamma-aminobutyrate transporter		61.59	49.87	51.23	43.65	155.17
ACICU_02438	methylmalonate-semialdehyde dehydrogenase		62.62	53.99	72.58	62.74	211.45
ACICU_02439	omega amino acid--pyruvate aminotransferase		86.91	128.15	133.39	91.07	431.80
ACICU_02488	hypothetical protein	2	259.14	627.17	141.11	131.88	127.17
ACICU_02501	ABC transporter substrate-binding protein		21.63	26.11	94.08	130.04	14.17
ACICU_02654	phosphonate ABC transporter substrate-l	3	558.35	854.55	305.55	237.18	731.01
ACICU_02695	membrane protein	2	128.39	92.00	61.59	93.98	34.89
ACICU_02696	membrane protein	2	460.49	383.65	276.01	420.25	146.28
ACICU_02780	permease	3	51.74	31.34	32.51	61.18	61.97
ACICU_02866	glycosyl transferase family 2	3	4.15	110.31	12.47	5.55	3.85
ACICU_02868	dolichyl-phosphate-mannose-protein ma	3	24.46	132.89	13.14	14.63	32.38
ACICU_02894	hypothetical protein		107.16	77.92	71.94	91.03	136.42
ACICU_02895	peptide synthetase	4	54.64	703.21	69.81	71.05	60.15
ACICU_02907	diacylglycerol kinase	3	29.02	143.62	11.76	19.05	15.75
ACICU_02930	carbamoyl phosphate synthase large subunit		5591.48	8015.59	9875.43	8705.71	17209.35
ACICU_02938	type I secretion protein		9198.27	5851.56	25940.18	16941.02	7037.04
ACICU_03000	ammonium transporter	3	68.15	34.85	545.15	493.88	54.23
ACICU_03001	hypothetical protein	3	6.99	55.91	2.76	10.70	3.85
ACICU_03002	pmrB histidine kinase	3	208.51	696.16	192.34	214.31	183.25
ACICU_03003	pmrA transcriptional regulator	3	34.95	166.57	29.07	41.00	29.91
ACICU_03004	pmrC lipid A phosphoethanolamine trans	3	22.29	490.15	6.24	18.36	25.50
ACICU_03056	entericidin	3	1050.24	1393.51	433.35	521.55	385.62
ACICU_03111	peroxidase	4	1909.00	1017.31	675.08	856.32	393.93
ACICU_03132	hypothetical protein	2	119.22	24.68	30.42	21.55	24.02
ACICU_03139	hypothetical protein		541.26	126.44	89.16	70.55	107.22
ACICU_03209	acetyl-CoA carboxylase		42.62	43.84	71.18	23.51	14.15
ACICU_03286	transcriptional regulator		27.27	68.18	58.07	263.08	101.04
ACICU_03287	GTP-binding protein	2	186.66	293.86	380.03	1091.33	350.38
ACICU_03323	transporter		1.74	4.72	0.69	3.29	91.39
ACICU_03326	hypothetical protein		478.71	267.58	214.37	314.79	111.65
ACICU_03347	hypothetical protein		155.81	108.58	67.05	56.81	41.46

ACICU_03422	sulfate permease	4	411.39	442.06	273.81	209.18	122.51
ACICU_03426	acetyl-CoA hydrolase		5779.60	7900.97	4181.39	4330.99	254.40
ACICU_03447	hypothetical protein	4	648.13	304.84	329.77	379.36	172.39
ACICU_03476	succinate-semialdehyde dehydrogenase		1020.81	1008.76	1301.15	1084.67	6365.68
ACICU_03477	4-aminobutyrate aminotransferase		1093.62	1305.87	1535.81	1180.88	10938.15
ACICU_03479	gamma-aminobutyrate transporter		841.11	827.51	904.85	692.15	6962.24
ACICU_03493	membrane protein	2	725.21	1857.97	3019.81	1958.43	3681.67
ACICU_03496	acetate permease		1502.34	1759.94	1467.07	1090.45	80.30
ACICU_03497	membrane protein		94.04	85.08	61.62	66.73	6.35
ACICU_03610	fumarylacetoacetase	4	949.65	669.97	534.96	491.41	182.20
ACICU_03625	glucose dehydrogenase		452.25	189.50	230.30	328.09	96.03
ACICU_03627	membrane protein	3	402.24	389.79	168.85	248.99	117.93

Patient 81

Locus Tag	Predicted Protein	DE in # of other patients	ABUH81366	ABUH81389	ABUH81452
ACICU_00740	5'-methylthioadenosine nucleosidase		43.33	121.03	183.56
ACICU_00762	trehalose phosphatase	6	49.93	97.28	230.62
ACICU_01027	hypothetical protein	4	198.61	367.74	915.78
ACICU_01071	TonB-dependent receptor		54.77	93.93	71.66
ACICU_01075	integrase		199.35	1025.54	1089.93
ACICU_01252	4-hydroxybenzoate transporter		13.29	124.42	168.57
ACICU_01470	transcriptional regulator	2	99.54	90.62	112.06
ACICU_01518	hypothetical protein	5	105.19	728.04	718.61
ACICU_01552	hypothetical protein	5	19.24	223.98	331.16
ACICU_01667	acyl-CoA dehydrogenase		101.79	309.02	409.14
ACICU_01668	acyl-CoA dehydrogenase		75.48	327.98	422.13
ACICU_01805	hypothetical protein	4	51.85	85.81	109.49
ACICU_01823	adeC putative outer meml	2	264.90	39.53	30.30
ACICU_01824	adeB cation/multidrug effl	2	1271.79	51.83	49.88
ACICU_01825	adeA membrane fusion pr	3	406.66	17.00	16.98
ACICU_01936	acetoacetate decarboxyla:	2	128.18	602.82	649.47
ACICU_01937	aldehyde dehydrogenase		110.20	456.85	558.31
ACICU_01953	GntR family transcriptional regulator		318.74	83.84	106.24
ACICU_01954	hypothetical protein		587.13	70.43	64.36
ACICU_01972	Rieske (2Fe-2S) protein	2	15.21	67.93	85.15
ACICU_01976	glutamyl-tRNA amidotrans	3	86.11	205.46	205.87
ACICU_02989	adenine deaminase		1272.40	5135.68	4638.12
ACICU_03004	pmrC lipid A phosphoetha	3	23.34	94.04	82.94
ACICU_03315	membrane protein		63.82	324.38	235.42
ACICU_03609	aromatic amino acid trans	2	35.22	890.20	900.85
ACICU_03610	fumarylacetoacetase	4	212.49	13145.48	10351.75
ACICU_03612	glyoxalase	2	17.47	202.21	186.58
ACICU_03614	4-hydroxyphenylpyruvate	3	127.93	5119.15	5471.34
RP84_13035	molecular chaperone DnaJ		4.01	71.31	44.28
RP84_13045	hypothetical protein		10.72	75.32	65.53
RP84_13050	hypothetical protein		79.70	504.13	589.86
RP84_13640	hypothetical protein		7.68	56.47	55.88
RP84_13645	hypothetical protein		9.45	49.01	66.35
RP84_13650	hypothetical protein		15.18	135.12	123.69
RP84_15225	hypothetical protein		23.32	254.75	229.71
RP84_15230	hypothetical protein		12.62	168.93	92.87
RP84_15235	hypothetical protein		18.61	112.48	121.06
RP84_15240	hypothetical protein		19.96	108.11	104.93
RP84_15245	hypothetical protein		7.47	55.61	46.43
RQ03_19675	hypothetical protein		0.42	32.93	35.06
RQ03_19700	membrane protein		81.98	409.02	335.45

Patient 280

Locus Tag	Predicted Protein	DE in # of other patients	ABUH28081	ABUH28092	ABUH28093	ABUH28099
ACICU_00024	membrane protein		183.29	321.54	302.22	834.78
ACICU_00035	hypothetical protein		237.79	59.48	40.34	48.24
ACICU_00083	hypothetical protein		286.26	415.67	404.01	331.30
ACICU_00228	dihydropteroate synthase		821.00	980.70	951.78	1197.38
ACICU_00684	membrane protein	4	230.73	74.39	79.60	66.75
ACICU_00741	hypothetical protein		95.08	153.45	174.54	420.04
ACICU_00761	trehalose-6-phosphate synthase	3	1574.45	188.64	178.62	103.85
ACICU_00762	trehalose phosphatase	6	135.09	15.34	11.82	4.97
ACICU_00895	hypothetical protein	3	183.33	18.65	8.71	10.64
ACICU_00900	polysaccharide deacetylase	3	482.46	968.13	963.54	4064.21
ACICU_00901	N-glycosyltransferase	3	580.01	752.05	723.96	2213.52
ACICU_00902	poly-beta-1,6-N-acetyl-D-glucosamine biosynth	2	190.83	418.05	319.71	976.08
ACICU_00989	CipA hypothetical protein		4370.76	2439.45	2745.86	904.08
ACICU_01003	ATPase AAA		31.65	92.66	54.50	46.18
ACICU_01198	cysteine-rich helical bundle repeat protein	4	2826.16	1019.86	1053.07	735.41
ACICU_01214	hypothetical protein	3	737.65	223.77	136.64	51.13
ACICU_01215	hypothetical protein	3	106.49	15.24	19.52	5.01
ACICU_01216	head morphogenesis protein	3	225.78	49.02	53.17	19.46
ACICU_01253	hypothetical protein		15.83	114.46	111.13	126.28
ACICU_01280	alcohol dehydrogenase	4	144.50	10.52	14.19	6.69
ACICU_01425	hypothetical protein	3	3194.35	757.10	653.20	231.30
ACICU_01426	hydroperoxidase	3	11358.19	2644.17	3174.74	1719.65
ACICU_01514	anthranilate synthase component II		193.55	447.06	800.41	412.96
ACICU_01518	hypothetical protein	5	411.94	649.52	856.19	3186.35
ACICU_01552	hypothetical protein	5	71.08	566.03	562.68	2939.18
ACICU_01553	hypothetical protein	4	25.35	46.63	35.95	190.39
ACICU_01710	hypothetical protein	2	1014.25	334.71	319.33	241.99
ACICU_01717	hypothetical protein		56.12	26.03	26.17	9.49
ACICU_01801	hypothetical protein	3	54.68	13.72	15.12	4.41
ACICU_01911	hemagglutinin	3	426.73	2731.51	2283.03	2544.38
ACICU_01912	hemolysin activator protein	4	97.31	963.46	748.41	1124.78
ACICU_02040	cytochrome d ubiquinol oxidase subunit 2	2	3121.33	6905.46	8440.21	10989.49
ACICU_02071	hypothetical protein	4	148.76	17.68	21.78	8.29
ACICU_02269	DNA breaking-rejoining protein	2	37.64	2.87	4.20	3.37
ACICU_02270	hypothetical protein	3	632.01	115.55	125.67	101.83
ACICU_02274	hypothetical protein		41.73	12.80	7.32	3.28
ACICU_02275	hypothetical protein	2	115.64	35.79	23.36	15.65
ACICU_02276	hypothetical protein	4	323.75	32.21	18.69	3.92
ACICU_02406	transporter	2	336.56	150.89	168.28	81.43
ACICU_02407	aspartate aminotransferase	2	360.64	179.40	223.51	105.58
ACICU_02431	DNA-binding protein	3	560.24	82.85	86.42	41.06
ACICU_02432	glycosyl transferase	3	91.93	20.22	33.16	29.38
ACICU_02433	methyltransferase	3	64.82	12.31	7.48	10.64
ACICU_02434	LmbE protein	3	100.90	22.66	14.79	23.82
ACICU_02435	acyl-CoA dehydrogenase	3	90.87	16.65	19.36	9.97
ACICU_02436	hypothetical protein	3	2237.99	400.91	378.42	335.88
ACICU_02462	cold-shock protein		4735.85	3306.29	2884.37	1376.38
ACICU_02527	membrane protein	2	441.31	91.20	88.49	70.59
ACICU_02697	glycosyl transferase	2	782.77	349.30	280.38	239.77
ACICU_02698	hypothetical protein	2	518.10	245.41	174.60	155.91
ACICU_02780	permease	3	45.16	50.10	73.82	72.42
ACICU_02865	hypothetical protein	2	43.33	77.54	58.52	332.90
ACICU_02866	glycosyl transferase family 2	3	11.68	34.97	27.85	388.76
ACICU_02867	polysaccharide biosynthesis protein GtrA	2	0.72	4.07	6.36	60.10
ACICU_02868	dolichyl-phosphate-mannose-protein mannosyl	3	24.24	33.02	28.80	293.49
ACICU_02895	peptide synthetase	4	107.93	409.81	411.70	2646.47

ACICU_02907	diacylglycerol kinase	3	24.18	78.78	73.84	291.83
ACICU_03001	hypothetical protein	3	7.25	18.76	30.88	129.98
ACICU_03002	pmrB histidine kinase	3	163.14	318.82	364.25	1457.18
ACICU_03003	pmrA transcriptional regulator	3	40.01	87.95	86.33	447.03
ACICU_03004	pmrC lipid A phosphoethanolamine transferase	3	43.39	230.55	207.60	1450.73
ACICU_03056	entericidin	3	1177.17	336.17	433.98	352.07
ACICU_03111	peroxidase	4	1356.31	425.80	342.21	275.14
ACICU_03287	GTP-binding protein	2	298.66	295.07	1154.12	676.23
ACICU_03305	DEAD/DEAH box helicase	3	56817.89	52598.46	60091.13	29797.75
ACICU_03382	hypothetical protein		124.63	94.45	71.82	116.77
ACICU_03394	pilus assembly protein PilM		354.49	1239.47	1068.93	1296.71
ACICU_03432	hypothetical protein		187.77	603.98	674.25	617.39
ACICU_03447	hypothetical protein	4	276.00	52.60	58.77	61.14
ACICU_03484	MFS transporter		506.10	945.52	823.13	1960.56
ACICU_03514	membrane protein	2	6573.58	2515.17	2151.43	1943.72
ACICU_03627	membrane protein	3	375.61	82.09	69.11	41.62
RP84_13880	conjugal transfer protein TraL		18.07	118.01	25.35	16.85
RP84_13885	hypothetical protein		28.57	146.06	28.59	25.00
RQ07_21540	acetylornithine aminotransferase		68.96	86.28	7.29	29.47

Patient 315

Locus Tag	Predicted Protein	DE in # of other patients	ABUH315100	ABUH315101
ACICU_00061	oxidoreductase	3	35.50	127.92
ACICU_00062	fatty acid desaturase	3	83.11	208.10
ACICU_00123	amino acid dehydrogenase		1160.68	2533.66
ACICU_00124	alanine racemase		853.23	1280.98
ACICU_00557	hypothetical protein	3	327.89	101.42
ACICU_00762	trehalose phosphatase	6	29.02	165.51
ACICU_00771	hypothetical protein		62.99	101.85
ACICU_00895	hypothetical protein	3	15.74	118.31
ACICU_01280	alcohol dehydrogenase	4	44.65	140.81
ACICU_01423	hypothetical protein	4	1067.71	5279.61
ACICU_01447	serine acetyltransferase		119.57	49.13
ACICU_01470	transcriptional regulator	2	54.70	189.95
ACICU_01471	hypothetical protein		3.40	39.03
ACICU_01472	ubiquinol oxidase subunit II		122.46	2934.33
ACICU_01473	cytochrome D ubiquinol oxidase subunit I		121.84	4010.43
ACICU_01474	hypothetical protein	2	310.67	2692.64
ACICU_01475	monooxygenase		211.00	1002.98
ACICU_01799	peptidase		449.84	270.19
ACICU_01869	ribose-phosphate pyrophosphokinase		49.90	83.40
ACICU_01919	amidase		2802.75	1866.40
ACICU_03585	hypothetical protein		4642.87	1245.96
ACICU_03598	formimidoylglutamase		73.86	182.92
ACICU_03609	aromatic amino acid transporter	2	49.10	107.24
ACICU_03610	fumarylacetoacetase	4	351.46	952.46
ACICU_03611	maleylacetoacetate isomerase		89.33	234.77
ACICU_03612	glyoxalase	2	41.03	129.39
ACICU_03614	4-hydroxyphenylpyruvate dioxygenase	3	183.50	428.94
RP84_02625	terminase		13.61	41.47
RP84_02630	hypothetical protein		6.33	18.08
RP84_02635	hypothetical protein		2.45	12.16
RP84_02640	phage capsid protein		19.93	115.02
RP84_02645	hypothetical protein		6.88	20.97
RP84_02675	hypothetical protein		17.74	143.05

Patient 348

Locus Tag	Predicted Protein	DE in # of other patients	ABUH34813	ABUH34827
ACICU_00093	L-lactate permease	2	4161.74	119.27
ACICU_00094	hypothetical protein	2	1036.09	67.17
ACICU_00095	lactate dehydrogenase	3	2433.22	160.84
ACICU_00096	lactate dehydrogenase	2	1361.05	150.81
ACICU_00265	iron transporter		412.29	116.41
ACICU_00266	iron transporter FeoB		4027.76	1277.78
ACICU_00463	biopolymer transporter ExbB		1475.56	384.00
ACICU_00485	ligand-gated channel protein		1050.09	237.06
ACICU_00557	hypothetical protein	3	109.42	380.33
ACICU_00746	hypothetical protein		16.09	200.68
ACICU_00747	heme utilization protein		79.53	472.62
ACICU_00876	hypothetical protein		43.49	5.24
ACICU_00942	outer membrane receptor protein		85.05	10.74
ACICU_01101	TonB-dependent receptor		358.87	100.28
ACICU_01109	hypothetical protein	2	70.56	257.00
ACICU_01121	hypothetical protein		14.07	75.22
ACICU_01271	acetyl-CoA carboxylase	2	1022.13	3313.12
ACICU_01423	hypothetical protein	4	1117.22	3712.87
ACICU_01518	hypothetical protein	5	1661.17	7298.17
ACICU_01552	hypothetical protein	5	306.70	2054.96
ACICU_01553	hypothetical protein	4	30.90	159.63
ACICU_01672	siderophore biosynthesis protein		2067.48	74.65
ACICU_01673	ornithine monooxygenase	2	857.95	31.47
ACICU_01674	RND transporter	2	724.85	41.83
ACICU_01675	siderophore achromobactin biosy	2	892.79	143.04
ACICU_01676	siderophore biosynthesis protein,	2	2289.10	174.88
ACICU_01677	(2Fe-2S)-binding protein	2	95.11	14.21
ACICU_01678	dimethylmenaquinone methyltrar	2	123.44	25.03
ACICU_01679	ligand-gated channel protein	2	10836.93	2145.19
ACICU_01681	peptidase	2	499.58	125.82
ACICU_01683	siderophore biosynthesis protein	2	1587.77	245.57
ACICU_01727	von Willebrand factor A		1094.31	45.70
ACICU_01728	tryptophan synthase subunit beta		279.90	64.70
ACICU_01790	2,3-dihydro-2,3-dihydroxybenzoate dehydrogena		127.05	8.83
ACICU_01791	2,3-dihydro-2,3-dihydroxybenzoate synthetase		235.05	44.77
ACICU_02262	magnesium ABC transporter ATPase		505.69	127.86
ACICU_02272	porin		89.56	18.20
ACICU_02408	membrane protein	2	6.39	25.78
ACICU_02414	protein CsuE	3	67.52	6.31
ACICU_02415	protein CsuD	3	45.78	3.92
ACICU_02416	protein CsuC	3	66.47	4.04
ACICU_02417	protein CsuB	3	31.01	1.18
ACICU_02418	protein CsuA	3	283.39	12.35
ACICU_02420	protein CsuA/B	3	3461.91	5.41
ACICU_02421	TetR family transcriptional regulator		75.54	0.50
ACICU_02485	hemin transporter HemP		166.96	41.37
ACICU_02570	isochorismate synthase		137.19	26.08

ACICU_02572	thioesterase		44.14	5.90
ACICU_02573	ABC transporter		202.68	34.88
ACICU_02574	ABC transporter		131.88	20.12
ACICU_02576	histidine decarboxylase		116.25	18.20
ACICU_02577	isochorismatase		69.08	8.88
ACICU_02578	enterobactin synthase subunit E		212.01	23.55
ACICU_02579	peptide synthetase		464.96	92.68
ACICU_02580	Rhizobactin siderophore biosynthesis protein Rhb		119.48	25.55
ACICU_02581	ligand-gated channel protein		352.56	52.53
ACICU_02583	iron ABC transporter ATP-binding protein		28.48	1.47
ACICU_02584	iron ABC transporter permease		42.20	7.39
ACICU_02585	iron ABC transporter permease		43.44	2.96
ACICU_02587	acinetobactin biosynthesis protein		338.35	33.33
ACICU_02588	peptide synthetase		295.23	64.31
ACICU_02589	acinetobactin utilization protein		91.72	11.30
ACICU_02895	peptide synthetase	4	623.66	2449.56
ACICU_03051	membrane protein		413.68	81.05
ACICU_03521	TonB-dependent receptor		657.70	198.29
ACICU_03537	ligand-gated channel protein		706.46	90.41

Patient 410

Locus Tag	Predicted Protein	DE in # of other patients	ABUH41096	ABUH410103	ABUH410128
ACICU_00061	oxidoreductase	3	110.53	18.48	103.03
ACICU_00062	fatty acid desaturase	3	224.72	42.23	166.23
ACICU_00093	L-lactate permease	2	941.16	49.25	701.15
ACICU_00094	hypothetical protein	2	144.00	23.75	86.90
ACICU_00095	lactate dehydrogenase	3	845.58	93.89	657.87
ACICU_00096	lactate dehydrogenase	2	342.86	76.95	244.76
ACICU_00318	signal peptide-containing protein		236.97	841.78	167.54
ACICU_00543	enoyl-ACP reductase		1670.30	7681.87	1736.95
ACICU_00594	acyl-CoA synthetase	2	189.63	931.76	199.62
ACICU_00608	hypothetical protein		383.31	96.48	306.78
ACICU_00612	acetyl-CoA carboxylase		1732.17	7967.36	1554.10
ACICU_00613	tRNA(Ile)-lysidine synthetase		67.03	278.91	74.95
ACICU_00634	aminobenzoate synthetase		48.16	30.48	42.67
ACICU_00684	membrane protein	4	7726.14	13996.49	3453.64
ACICU_00699	hypothetical protein		166.48	243.35	207.50
ACICU_00762	trehalose phosphatase	6	442.68	1817.25	376.68
ACICU_00775	ACP S-malonyltransferase		1352.74	4136.58	1375.29
ACICU_00776	3-oxoacyl-ACP reductase		2027.13	6614.09	2105.02
ACICU_00801	LysR family transcriptional regulator		29.90	86.74	34.57
ACICU_00803	transporter		77.47	226.79	97.08
ACICU_00804	lipase		25.19	73.50	24.21
ACICU_00813	3-oxoacyl-ACP synthase		3580.61	11930.16	3521.67
ACICU_00888	choline dehydrogenase	3	2064.73	915.62	563.67
ACICU_01027	hypothetical protein	4	395.95	2376.17	446.16
ACICU_01085	amino acid transporter	3	50.34	39.04	128.31
ACICU_01102	ribonuclease T		888.58	272.46	848.51
ACICU_01103	dihydroorotase		1317.27	410.41	1229.96
ACICU_01116	type VI secretion system protein		1967.82	1637.53	2044.13
ACICU_01135	acetylornithine aminotransferase		26.88	4.28	32.82
ACICU_01136	arginine N-succinyltransferase		24.07	5.11	31.76
ACICU_01163	acyltransferase		11.46	46.39	13.22
ACICU_01165	protein disaggregation chaperone		5795.53	19410.76	8685.27
ACICU_01166	damage-inducible protein CinA		65.19	237.52	57.00
ACICU_01167	hypothetical protein		30.86	104.10	36.76
ACICU_01169	hypothetical protein		13.30	44.28	16.82
ACICU_01177	glutathione S-transferase		1143.15	3006.65	866.55
ACICU_01179	NADH dehydrogenase		1210.84	802.90	502.39
ACICU_01180	hypothetical protein		12.35	80.52	11.82
ACICU_01181	methyltransferase		152.90	1019.24	186.89
ACICU_01182	lysine tRNA synthetase		15.22	94.04	27.55
ACICU_01186	benzoate transporter		12.33	40.17	16.99
ACICU_01198	cysteine-rich helical bundle repeat protein	4	5425.64	25340.09	5678.21
ACICU_01199	hypothetical protein		132.82	470.63	345.29
ACICU_01221	hypothetical protein	3	1622.68	5428.87	1070.70
ACICU_01222	glycine zipper	2	1068.91	2902.66	869.15
ACICU_01261	type VI secretion system protein	3	1453.41	1184.45	1207.69
ACICU_01280	alcohol dehydrogenase	4	391.36	1857.81	314.99
ACICU_01335	enoyl-CoA hydratase		157.87	3756.35	263.44
ACICU_01336	ATPase AAA		259.28	9968.04	586.70
ACICU_01337	phenylacetate-CoA oxygenase		17.54	620.34	33.57
ACICU_01338	phenylacetic acid degradation protein		133.60	4888.35	236.80
ACICU_01339	phenylacetate-CoA oxygenase		105.25	4325.21	238.07

ACICU_01340	phenylacetic acid degradation protein		80.88	2365.92	123.71
ACICU_01341	2,3-dehydroadipyl-CoA hydratase		24.72	526.92	29.46
ACICU_01342	enoyl-CoA hydratase		76.73	925.95	91.24
ACICU_01343	3-hydroxyacyl-CoA dehydrogenase		76.21	1459.09	124.49
ACICU_01344	beta-ketoadipyl CoA thiolase		49.08	652.52	68.75
ACICU_01345	phenylacetate--CoA ligase	2	91.87	573.11	93.43
ACICU_01404	thioesterase		74.63	801.19	61.11
ACICU_01405	MFS transporter		3175.20	10423.55	3778.76
ACICU_01406	LysR family transcriptional regulator		20.97	102.92	31.85
ACICU_01419	hypothetical protein		14.20	63.88	3.02
ACICU_01424	damage-inducible protein CinA		14.30	61.91	12.04
ACICU_01427	short-chain dehydrogenase	3	1413.51	2908.48	854.53
ACICU_01428	hypothetical protein		15.72	183.56	18.95
ACICU_01430	hypothetical protein		29.11	92.56	24.68
ACICU_01541	LysR family transcriptional regulator		40.90	149.41	37.85
ACICU_01554	biotin synthase		780.85	2252.06	678.45
ACICU_01571	hypothetical protein		74.88	183.70	36.32
ACICU_01585	ArsR family transcriptional regulator		24.37	37.69	7.29
ACICU_01625	AraC family transcriptional regulator		83.21	400.36	72.11
ACICU_01626	ATPase		1764.35	4344.81	1480.86
ACICU_01670	hypothetical protein		62.32	194.02	66.35
ACICU_01673	ornithine monooxygenase	2	196.35	46.51	31.63
ACICU_01674	RND transporter	2	142.08	49.69	32.90
ACICU_01675	siderophore achromobactin biosynthesis protein AcsC	2	680.65	199.19	145.86
ACICU_01676	siderophore biosynthesis protein, IucA/IucC family	2	928.10	195.60	206.50
ACICU_01677	(2Fe-2S)-binding protein	2	104.70	27.13	10.97
ACICU_01678	dimethylmenaquinone methyltransferase	2	184.33	28.31	34.92
ACICU_01679	ligand-gated channel protein	2	4987.20	449.47	462.00
ACICU_01680	hypothetical protein		24.67	3.65	3.78
ACICU_01681	peptidase	2	519.15	76.07	77.13
ACICU_01682	hypothetical protein		52.34	11.49	7.44
ACICU_01683	siderophore biosynthesis protein	2	4419.32	109.42	80.95
ACICU_01684	haloacid dehalogenase		6750.51	2144.09	961.45
ACICU_01708	hypothetical protein		526.14	1434.53	381.15
ACICU_01805	hypothetical protein	4	113.32	310.02	77.42
ACICU_01813	pilus assembly protein fimA	2	197.83	79.49	441.67
ACICU_01825	adeA membrane fusion protein	3	529.79	156.19	354.78
ACICU_01912	hemolysin activator protein	4	123.54	38.11	156.12
ACICU_02071	hypothetical protein	4	161.78	574.86	138.12
ACICU_02088	UDP-N-acetylglucosamine acyltransferase		1798.03	5047.68	1724.59
ACICU_02089	3-hydroxyacyl-ACP dehydratase		1045.03	4248.45	884.39
ACICU_02135	3-dehydroquinate dehydratase		84.42	227.72	55.96
ACICU_02276	hypothetical protein	4	330.14	1038.01	169.63
ACICU_02289	hypothetical protein	2	133.33	595.01	181.84
ACICU_02359	hypothetical protein		67.92	142.58	244.57
ACICU_02381	hypothetical protein		141.95	424.46	147.94
ACICU_02382	hypothetical protein		273.61	703.36	314.89
ACICU_02414	protein CsuE	3	48.93	9.72	238.53
ACICU_02415	protein CsuD	3	50.13	9.76	211.13
ACICU_02416	protein CsuC	3	75.42	19.15	348.73
ACICU_02417	protein CsuB	3	66.76	21.14	242.33
ACICU_02418	protein CsuA	3	132.44	45.37	1107.43
ACICU_02420	protein CsuA/B	3	1093.49	188.06	4190.64
ACICU_02538	RNA methyltransferase		156.57	57.71	124.07
ACICU_02653	ABC transporter permease		234.33	83.49	276.64

ACICU_02654	phosphonate ABC transporter substrate-binding protein	3	244.86	69.86	298.67
ACICU_02655	aromatic amino acid transporter		14.28	149.86	623.82
ACICU_02656	pyruvate decarboxylase		126.28	3964.05	286.76
ACICU_02657	AsnC family transcriptional regulator		21.40	106.76	25.86
ACICU_02658	aldehyde dehydrogenase		1036.93	6063.72	1384.53
ACICU_03000	ammonium transporter	3	106.80	43.04	163.45
ACICU_03007	acyltransferase		64.21	381.62	96.30
ACICU_03111	peroxidase	4	1468.43	3968.29	1507.26
ACICU_03116	acetyl-CoA carboxyl transferase		1667.98	4572.37	1817.98
ACICU_03117	tryptophan synthase subunit alpha		1923.65	5118.40	2012.47
ACICU_03305	DEAD/DEAH box helicase	3	48506.38	15940.68	44948.25
ACICU_03337	hypothetical protein		337.20	1470.80	307.38
ACICU_03422	sulfate permease	4	253.60	420.27	157.92
ACICU_03447	hypothetical protein	4	415.08	1211.43	547.93
ACICU_03493	membrane protein	2	1867.96	548.55	2498.35
ACICU_03499	hypothetical protein		1338.57	10853.84	1435.36
ACICU_03602	urocanate hydratase		1181.45	664.84	2625.06

Patient 475

Locus Tag	Predicted Protein	DE in # of other patients	ABUH475197	ABUH475239	ABUH475361
ACICU_00106	membrane protein		33.54	75.68	78.63
ACICU_00115	short-chain dehydrogenase		3.47	28.20	6.68
ACICU_00126	amino acid transporter		325.28	1057.97	740.46
ACICU_00164	malate dehydrogenase		1204.24	3451.77	2051.53
ACICU_00166	threonine transporter RhtB		75.22	147.15	83.46
ACICU_00312	heat shock protein 90		457.98	1456.85	1198.13
ACICU_00319	hypothetical protein		41.24	14.33	63.48
ACICU_00345	general secretion pathway protein GspE		121.03	382.23	150.86
ACICU_00494	phosphogluconate dehydratase		3117.79	1291.55	4286.18
ACICU_00495	2-dehydro-3-deoxyphosphogluconate aldolase		1149.54	372.58	1960.77
ACICU_00496	gluconate permease		1989.21	645.84	2875.81
ACICU_00498	aldehyde dehydrogenase		2080.72	622.92	2939.91
ACICU_00557	hypothetical protein	3	62.87	21.92	141.22
ACICU_00571	NAD(P) transhydrogenase subunit alpha	2	558.06	128.59	250.25
ACICU_00684	membrane protein	4	230.09	112.40	952.72
ACICU_00738	hypothetical protein		4404.70	943.87	4356.16
ACICU_00760	ABC transporter permease		1012.76	234.57	663.57
ACICU_00761	trehalose-6-phosphate synthase	3	2027.08	85.98	1522.33
ACICU_00762	trehalose phosphatase	6	99.77	2.57	49.89
ACICU_00861	gamma-glutamyltransferase	2	236.95	150.43	234.33
ACICU_00888	choline dehydrogenase	3	9317.77	885.73	1888.35
ACICU_00889	betaine-aldehyde dehydrogenase	2	11644.49	1682.94	3344.85
ACICU_00890	BetI family transcriptional regulator	2	2263.86	574.01	781.37
ACICU_00891	choline transporter	2	1763.25	345.00	623.84
ACICU_00895	hypothetical protein	3	133.56	57.25	239.32
ACICU_00969	copper resistance protein NlpE		4618.98	1198.21	3285.10
ACICU_01023	hypothetical protein		92.11	62.42	0.00
ACICU_01027	hypothetical protein	4	77.17	14.20	0.00
ACICU_01035	head morphogenesis protein		39.00	33.44	9.53
ACICU_01085	amino acid transporter	3	42.01	142.60	41.21
ACICU_01109	hypothetical protein	2	107.12	30.42	152.04
ACICU_01119	Sel1 repeat protein		139.38	66.78	229.09
ACICU_01172	membrane protein		556.68	578.12	2537.02
ACICU_01193	LysR family transcriptional regulator		342.49	142.70	110.46
ACICU_01194	heavy metal transporter		233.75	96.56	67.73
ACICU_01195	ATPase		1604.53	562.63	481.21
ACICU_01198	cysteine-rich helical bundle repeat protein	4	4256.00	703.07	2645.40
ACICU_01210	cold-shock protein		162.90	4.18	32.94
ACICU_01214	hypothetical protein	3	508.74	70.09	150.38
ACICU_01215	hypothetical protein	3	132.22	8.12	100.96
ACICU_01216	head morphogenesis protein	3	215.08	13.07	153.00
ACICU_01221	hypothetical protein	3	350.96	8.75	155.51
ACICU_01222	glycine zipper	2	191.12	32.67	153.45
ACICU_01260	beta-lactamase		86.83	30.57	113.19
ACICU_01261	type VI secretion system protein	3	475.43	246.88	522.61
ACICU_01266	membrane protein	2	546.90	3155.72	1144.42
ACICU_01267	LamB/YcsF family protein		310.19	1609.47	635.83
ACICU_01268	hypothetical protein	2	470.51	2647.33	1202.37
ACICU_01270	allophanate hydrolase	2	271.01	2139.74	655.12
ACICU_01271	acetyl-CoA carboxylase	2	855.58	4292.96	1611.48
ACICU_01280	alcohol dehydrogenase	4	142.74	11.72	55.87
ACICU_01322	hemolysin		260.55	80.19	108.28
ACICU_01345	phenylacetate--CoA ligase	2	230.29	1312.26	370.60
ACICU_01348	phenylacetic acid degradation protein		11.56	58.05	25.04
ACICU_01420	hypothetical protein	2	82.42	7.43	21.94
ACICU_01421	hypothetical protein	2	76.99	6.42	24.45
ACICU_01422	hypothetical protein	2	5481.71	27.66	1234.39

ACICU_01423	hypothetical protein	4	3801.38	21.49	868.53
ACICU_01425	hypothetical protein	3	3468.22	119.48	1271.90
ACICU_01426	hydroperoxidase	3	13638.11	504.34	8828.25
ACICU_01427	short-chain dehydrogenase	3	512.04	16.29	100.00
ACICU_01429	stress-induced protein		3946.64	49.22	788.20
ACICU_01474	hypothetical protein	2	478.62	152.37	161.20
ACICU_01498	NADH dehydrogenase		240.89	227.64	183.22
ACICU_01591	hypothetical protein		51.16	56.58	13.96
ACICU_01623	membrane protein		153.37	65.71	155.59
ACICU_01710	hypothetical protein	2	827.68	171.29	517.00
ACICU_01721	hypothetical protein		490.78	159.27	533.65
ACICU_01800	hypothetical protein		337.29	22.64	159.98
ACICU_01801	hypothetical protein	3	57.47	2.48	22.49
ACICU_01805	hypothetical protein	4	22.57	0.00	15.29
ACICU_01806	catalase		369.96	92.45	346.82
ACICU_01807	phage capsid and scaffold protein		300.27	94.65	377.06
ACICU_01911	hemagglutinin	3	467.32	1784.45	663.81
ACICU_01912	hemolysin activator protein	4	127.49	696.80	270.04
ACICU_01935	alpha/beta hydrolase	2	3226.77	1007.08	2936.00
ACICU_01936	acetoacetate decarboxylase	2	110.47	31.25	60.23
ACICU_01971	aromatic-ring-hydroxylating dioxygenase		5.86	38.15	4.58
ACICU_01972	Rieske (2Fe-2S) protein	2	37.92	94.34	22.79
ACICU_01975	acyl-CoA dehydrogenase	2	16.69	70.89	15.92
ACICU_01976	glutamyl-tRNA amidotransferase	3	103.74	196.98	58.46
ACICU_02040	cytochrome d ubiquinol oxidase subunit 2	2	3371.74	12764.95	8175.98
ACICU_02043	hypothetical protein		35.82	89.66	16.99
ACICU_02071	hypothetical protein	4	113.80	12.79	25.56
ACICU_02073	serine protease		319.11	76.12	369.64
ACICU_02080	hypothetical protein		58.70	9.28	31.28
ACICU_02192	hypothetical protein		37.84	36.76	0.00
ACICU_02193	hypothetical protein		30.18	36.41	0.00
ACICU_02196	hypothetical protein		33.54	24.31	0.00
ACICU_02236	hypothetical protein		708.72	216.02	1555.68
ACICU_02269	DNA breaking-rejoining protein	2	30.78	0.44	10.23
ACICU_02270	hypothetical protein	3	518.26	80.05	263.79
ACICU_02275	hypothetical protein	2	108.17	10.88	65.70
ACICU_02276	hypothetical protein	4	258.71	3.60	29.99
ACICU_02327	hypothetical protein		42.47	10.25	49.97
ACICU_02400	sorbosone dehydrogenase		443.89	118.90	278.86
ACICU_02406	transporter	2	371.61	24.37	235.58
ACICU_02407	aspartate aminotransferase	2	490.79	26.20	297.15
ACICU_02408	membrane protein	2	16.66	4.53	18.44
ACICU_02412	glycine/betaine ABC transporter		41.63	15.43	52.63
ACICU_02413	hypothetical protein		6.16	5.88	20.53
ACICU_02414	protein CsuE	3	16.75	5.45	18.85
ACICU_02415	protein CsuD	3	9.96	2.68	9.32
ACICU_02416	protein CsuC	3	10.49	3.27	9.55
ACICU_02417	protein CsuB	3	9.72	1.71	6.29
ACICU_02418	protein CsuA	3	43.22	5.62	35.26
ACICU_02420	protein CsuA/B	3	185.01	23.16	163.42
ACICU_02429	membrane protein		55.12	4.61	20.02
ACICU_02431	DNA-binding protein	3	535.01	32.29	167.85
ACICU_02432	glycosyl transferase	3	135.28	12.29	105.58
ACICU_02433	methyltransferase	3	63.37	10.02	65.40
ACICU_02434	LmbE protein	3	85.22	10.16	91.29
ACICU_02435	acyl-CoA dehydrogenase	3	65.70	11.66	55.06
ACICU_02436	hypothetical protein	3	1920.95	55.22	1224.18
ACICU_02527	membrane protein	2	468.20	108.62	295.71
ACICU_02566	hypothetical protein		1740.81	1220.45	3651.59
ACICU_02654	phosphonate ABC transporter substrate-binc	3	200.29	473.62	132.36

ACICU_02695	membrane protein	2	81.80	7.15	49.41
ACICU_02696	membrane protein	2	218.98	56.62	144.95
ACICU_02697	glycosyl transferase	2	616.69	174.91	422.67
ACICU_02698	hypothetical protein	2	425.82	102.70	305.49
ACICU_02783	hypothetical protein		62.30	202.27	111.42
ACICU_02910	hypothetical protein		2621.47	1075.14	1438.25
ACICU_02946	hemagglutinin		1527.93	653.46	2773.93
ACICU_02962	hypothetical protein		20.18	82.27	56.76
ACICU_02966	hypothetical protein		21.59	4.96	24.23
ACICU_03000	ammonium transporter	3	125.56	32.58	84.24
ACICU_03056	entericidin	3	756.72	234.94	553.29
ACICU_03061	twitching motility protein PilT		52.30	164.34	54.07
ACICU_03062	chemotaxis protein CheY		45.04	164.70	54.51
ACICU_03092	glyoxalase		111.26	22.70	151.16
ACICU_03111	peroxidase	4	1153.45	228.14	506.09
ACICU_03132	hypothetical protein	2	28.65	52.17	8.75
ACICU_03336	amino acid transporter		529.69	165.48	352.88
ACICU_03355	hemerythrin		540.47	97.79	418.57
ACICU_03418	Sel1 repeat protein		636.43	189.09	344.56
ACICU_03422	sulfate permease	4	190.73	55.80	372.96
ACICU_03447	hypothetical protein	4	348.55	87.97	409.67
ACICU_03514	membrane protein	2	5101.62	1558.20	2501.46
ACICU_03522	membrane protein		69.26	40.83	12.04
ACICU_03576	ABC transporter substrate-binding protein		173.84	18.56	159.58
ACICU_03605	acyltransferase		74.36	5.98	53.33
ACICU_03610	fumarylacetoacetase	4	493.28	366.08	121.65
ACICU_03614	4-hydroxyphenylpyruvate dioxygenase	3	195.05	414.21	111.09
ACICU_03627	membrane protein	3	267.53	29.50	94.65

Patient 588

Locus Tag	Predicted Protein	DE in # of other patients	ABUH588656	ABUH588663
ACICU_00400	50S ribosomal protein L31		152.85	1093.76
ACICU_00900	polysaccharide deacetylase	3	546.07	2243.68
ACICU_00901	N-glycosyltransferase	3	327.98	1164.10
ACICU_00902	poly-beta-1,6-N-acetyl-D-glucosamine I	2	97.79	446.81
ACICU_01072	lipid A phosphoethanolamine transferase		10.72	137.04
ACICU_01518	hypothetical protein	5	94.52	8068.90
ACICU_01552	hypothetical protein	5	13.98	5087.33
ACICU_01553	hypothetical protein	4	17.14	265.15
ACICU_02488	hypothetical protein	2	548.35	3528.93
ACICU_02552	peptidase M24		548.96	1846.54
ACICU_02780	permease	3	32.17	68.32
ACICU_02813	carO, membrane protein		1268.95	10391.19
ACICU_02865	hypothetical protein	2	46.79	218.88
ACICU_02866	glycosyl transferase family 2	3	6.05	280.17
ACICU_02867	polysaccharide biosynthesis protein Gt	2	0.99	45.68
ACICU_02868	dolichyl-phosphate-mannose-protein n	3	19.40	267.72
ACICU_02895	peptide synthetase	4	59.89	2817.19
ACICU_02907	diacylglycerol kinase	3	23.56	242.22
ACICU_03001	hypothetical protein	3	8.65	128.04
ACICU_03002	pmrB histidine kinase	3	274.07	959.10
ACICU_03003	pmrA transcriptional regulator	3	49.60	302.11
ACICU_03004	pmrC lipid A phosphoethanolamine tra	3	29.49	923.92
ACICU_03142	TonB-dependent receptor		145.78	882.83
ACICU_03305	DEAD/DEAH box helicase	3	6926.19	9866.88
ACICU_03422	sulfate permease	4	825.26	2828.73
ACICU_03608	peptidase M15		18.79	114.67
