# Supplementary material for: Transcriptome Remodeling of Acinetobacter baumannii during Infection and Treatment
Source: mBio. 2017 Mar 7;8(2):e02193-16. doi: 10.1128/mBio.02193-16 (PMC5340874; doi:10.1128/mBio.02193-16)
Supplement: TABLE S2 [file mbo001173221st2.pdf]

Table S2. SNV summary table

Patient Specific

Patient	Locus	Strains	Mutation	ACICU coordinate (CP000863.1)	Predicted Annotation
**66**	ACICU_01593	**ABUH66241**	D330V	1705134	predicted peptidylprolyl isomerase
	ACICU_01187		V435D	1292944	predicted MFS transporter
	IG		IG		
	ACICU_03134	**ABUH66253**	L435H	3336320	predicted acyl-CoA dehydrogenase
	ACICU_03653		E29A	3878791	hypothetical protein
	ACICU_03047		K95I	3237437	P-nitrobenzoate reductase NfnB
	ACICU_01827		D167N	1953791	histidine kinase AdeS
	ACICU_03002		S17R	3190434	PmrB
	ACICU_01157		A138G	1258634	predicted adenylosuccinate synthase
	ACICU_01739		A127V	1853419	predicted dehydrogenase
	ACICU_02177		Q85H	2316655	hypothetical protein
	ACICU_03477	**ABUH66276**	A254V	3684991	predicted 4-aminobutyrate transaminase
	ACICU_03495		Q164Stop	3706656	predicted ion channel membrane protein
	ACICU_03498		T57I	3709718	histidine kinase
	ACICU_00089		synonymous	97031	UDP-glucose 6-dehydrogenase, Ugd
	ACICU_01827		R313S	1954231	histidine kinase AdeS
	ACICU_01674		P72L	1788182	predicted MFS transporter
	ACICU_00303		D525V	327973	RNA polymerase, beta subunit RpoB
	ACICU_02683		synonymous	2853732	predicted MFS transporter
	ACICU_02085		A80T	2221632	RecA
	IG		IG		intergenic at 2272424
	ACICU_02039		L428Q	2179008	cytochrome bd-type quinol oxidase
	ACICU_02495		R197C	2634918	predicted protease
**81**	ACICU_00804	**ABUH81366**	synonymous	897417	synonymous at 897086
	ACICU_01826		A80P	1952756	response regulator AdeR
	ACICU_02988		Q729Stop	3174596	cation/multidrug efflux pump AdeJ
	ACICU_00681	**ABUH81389**	insertion, frameshift at 627AA	754646	predicted restriction endonuclease
	IG		IG		intergenic at 1499725
	ACICU_03326	**ABUH81452**	synonymous	3527672	predicted lipoprotein
**280**	ACICU_03002	**ABUH28081**	L94Q	3190204	PmrB
	ACICU_02317	**ABUH28093**	K127I	2445774	ribosomal small subunit pseudouridine synthase A RsuA
	ACICU_01889	**ABUH28099**	synonymous	2010317	predicted MFS transporter
	ACICU_03002		L257I	3189716	PmrB
**315**	ACICU_03157	**ABUH315100**	W261L	3360002	histidine kinase
	ACICU_01399		Q319H	1504884	predicted porin
	ACICU_00546	**ABUH315101**	synonymous	597683	MacA
	ACICU_01470		N90K	1579686	transcriptional regulator
**348**	V427_0076	**ABUH34813**	H128Y		hypothetical protein
	ACICU_00253		LR55C	274453	LPS export ABC transporter permease LptF
	ACICU_00266		L9P	289416	iron transporter FeoB
	ACICU_03230		K124Stop	3441077	hypothetical lipoprotein
	ACICU_01602	**ABUH34827**	K41N	1714042	lipid A export permease MsbA
	ACICU_01308		L303Q	1414709	ACICU_01308 L303Q T6SS inner membrane protein
	IG		IG		intergenic
**410**	none				
**475**	ACICU_03157	**ABUH475239**	G413Stop	3359547	histidine kinase
	ACICU_00071	**ABUH475361**	P541H	78574	Wcz chain length determinant, tyrosine kinase
	ACICU_01128		V338I	1225351	predicted glycine/D-amino acid oxidase
**588**	ACICU_02689	**ABUH588656**	T383S	2859017	predicted monomeric isocitrate dehydrogenase
	ACICU_01096		synonymous	1188586	predicted transporter
	ACICU_03484		A241V	3692163	predicted MFS transporter
	ACICU_00706		synonymous	793755	histidine kinase
	ACICU_00170	**ABUH588663**	P28L	193257	predicted periplasmic metal binding protein family
	ACICU_03002		E229D	3189798	PmrB
	ACICU_03002		Q247L	3189745	PmrB

Patient Restricted

Patient	Locus	Strains	Mutation	ACICU coordinate (CP000863.1)	Predicted Annotation
**66**	ACICU_00712	ABUH66253, ABUH66276	I82F	800157	NADH:ubiquinone oxidoreductase 24kD sub
	ACICU_00071	ABUH66268, ABUH66271	P542L	78574	Wcz chain length determinant
	ACICU_00410	ABUH66268, ABUH66271	L18F	453415	ribonuclease E/G Rne
	IG	ABUH66268, ABUH66271, ABUH66276	IG	2527549	intergenic
**81**	up ACICU_01825	ABUH81389, ABUH81452	5bp del at -40	1952412	deletion at -40 from AdeA
	ACICU_03189	ABUH81389, ABUH81452	L50Q	3396646	predicted phosphohydrolase
	ACICU_00166	ABUH81389, ABUH81452	A24V	190418	predicted amino acid efflux
	ACICU_01391	ABUH81389, ABUH81452	G310S	1495608	acetyl-CoA C-acetyltransferase
	ACICU_03002	ABUH81389, ABUH81452	T115I	3190141	PmrB
**280**	ACICU_03063	ABUH28092, ABUH28099	K114Stop	3257978	pilus assembly protein PilG
	ACICU_00705	ABUH28092, ABUH28093, ABUH28099	C70S	792284	transcriptional regulator
	ACICU_00545	ABUH28092, ABUH28093	V328W	595540	MacB ABC-type efflux pump
	ACICU_02581	ABUH28092, ABUH28093, ABUH28099	A100T	2737785	acinetobactin receptor BauA
	ACICU_03002	ABUH28092, ABUH28093, ABUH28099	V31F	3190394	PmrB
	ACICU_02130	ABUH28092, ABUH28099	G286V	2267550	nitrate reductase NirD
	ACICU_01494	ABUH28092, ABUH28099	I183N	1602946	arsenic resistance ArsB
	ACICU_01494	ABUH28092, ABUH28099	L191F	1602971	arsenic resistance ArsB
**410**	ACICU_03027	ABUH410103, ABUH410108	1bp deletion	3217412	hypothetical protein
	ACICU_03387	ABUH410103, ABUH410108	1bp deletion	3593806	signal peptide conserved hypothetical
