# Supplementary material for: Transcriptome Remodeling of Acinetobacter baumannii during Infection and Treatment
Source: mBio. 2017 Mar 7;8(2):e02193-16. doi: 10.1128/mBio.02193-16 (PMC5340874; doi:10.1128/mBio.02193-16)
Supplement: TABLE S3 [file mbo001173221st3.pdf]

Table S3. Isolate-specific insertion seqence summary

gene_before	gene_after	in_gene	orientation	element	ABUH588656	ABUH588663	ABUH34813	ABUH34827	ABUH66241	ABUH66253	ABUH66268	ABUH66271	ABUH66276	ABUH28081	ABUH28092	ABUH28093	ABUH28099	ABUH475197	ABUH475239	ABUH475361	ABUH315100	ABUH315101	ABUH41096	ABUH410103	ABUH410128	ABUH81366	ABUH81389	ABUH81452
ACICU_00086	ACICU_00087		R	IS*Aba*125									1															
ACICU_00288	ACICU_00289		R	IS*Aba*125															1									
ACICU_00288	ACICU_00289		F	IS*Aba*12				1																				
		ACICU_00555	F	IS*Aba*125			1																					
		ACICU_00745	R	IS*Aba*125			1																					
ACICU_01103	ACICU_01104		F	IS*Aba*1																				1				
ACICU_01551	ACICU_01552		F	IS*Aba*27																1								
		ACICU_01720	F	IS*Aba*125															1									
		ACICU_01765	F	IS*Aba*1									1															
ACICU_01839	ACICU_01840		R	IS*Aba*27		1																						
ACICU_02073	ACICU_02074		R	IS*Aba*27	1																							
ACICU_02243	ACICU_02244		R	IS*Aba*27		1																						
ACICU_02408	ACICU_02409		F	IS*Aba*125			1																					
ACICU_02420	ACICU_02421		F	IS*Aba*1																					1			
		ACICU_02421	F	IS*Aba*12				1																				
		ACICU_02538	F	IS*Aba*125																				1				
		ACICU_02613	R	IS*Aba*125														1										
ACICU_02685	ACICU_02686		F	IS*Aba*27		1																						
		ACICU_02813	R	IS*Aba*27	1																							
		ACICU_02910	R	IS*Aba*125																1								
		ACICU_03345	F	IS*Aba*1									1															
		ACICU_03355	R	IS*Aba*1																					1			
		ACICU_03472	R	IS*Aba*125																				1				
		ACICU_03611	F	IS*Aba*125																							1	1
