# Supplementary material for: Transcriptome Remodeling of Acinetobacter baumannii during Infection and Treatment
Source: mBio. 2017 Mar 7;8(2):e02193-16. doi: 10.1128/mBio.02193-16 (PMC5340874; doi:10.1128/mBio.02193-16)
Supplement: TABLE S4 [file mbo001173221st4.pdf]

Table S4. Gene identified as differentially expressed among clades.

			Clade	A												B					C					D	
Locus Tag	predicted annotation	specific genetic event	type of change	ABUH315100	ABUH315101	ABUH28081	ABUH28092	ABUH28093	ABUH28099	ABUH475197	ABUH475239	ABUH475361	ABUH81366	ABUH81389	ABUH81452	ABUH66241	ABUH66253	ABUH66268	ABUH66271	ABUH66276	ABUH41096	ABUH410103	ABUH410128	ABUH588656	ABUH588663	ABUH34813	ABUH34827
ACICU_00306	hypothetical protein			1.5	0.7	1.6	1.3	0.0	0.0	0.4	1.7	2.2	5.8	10.4	7.0	3.0	1.2	5.8	1.7	1.2	8.9	7.6	12.4	19.7	41.9	17.2	21.5
ACICU_00307	hot-dog fold hypothetical protein	ISAba1 in ACICU_00307, clade C	insertion sequence	47.8	62.0	35.2	48.4	49.1	52.4	40.4	26.8	43.1	84.2	73.7	67.7	45.1	47.9	50.0	73.5	39.4	420.2	392.5	468.1	463.2	415.7	52.1	48.0
ACICU_00384	tRNA-binding protein	none ID		136.9	98.2	93.5	99.1	91.3	107.0	127.1	62.2	131.5	244.1	220.6	266.5	172.9	155.5	130.7	225.2	257.1	135.6	183.3	304.2	193.9	195.0	425.6	584.3
ACICU_00450	PF03729 repeat protein	none ID		44.8	67.0	44.3	31.9	27.2	29.3	37.5	25.2	28.0	67.6	156.4	187.0	216.9	70.5	58.5	82.8	120.9	82.0	109.5	183.3	192.4	233.8	342.7	356.4
ACICU_00471	prolipoprotein diacylglyceryl transferase	none ID		27.3	34.7	36.7	29.1	25.9	33.0	25.2	52.9	32.3	48.0	62.3	68.2	27.2	30.5	36.7	21.8	26.8	120.5	90.4	99.6	118.6	95.6	108.3	104.5
ACICU_00545	macrolide transporter			51.5	45.6	39.6	49.7	49.9	56.6	42.3	39.9	57.5	37.0	39.0	37.4	117.0	46.1	54.5	49.0	55.3	35.8	28.6	40.5	33.3	57.7	10.7	8.0
ACICU_00546	secretion protein HylD	ISAba2 in clade D	insertion sequence	58.0	58.8	36.4	56.2	51.1	60.5	54.3	49.6	54.0	56.0	47.5	46.0	108.7	50.1	49.3	50.8	63.8	45.9	39.6	54.6	51.2	61.0	22.8	29.8
ACICU_00684	membrane protein	clade C has G to A @761268, start	intergenic mutation	26.4	32.2	19.3	6.6	7.3	6.4	19.7	10.5	95.0	299.5	242.3	327.8	113.1	68.6	62.9	45.1	8.2	707.4	1250.5	351.6	421.5	931.1	147.4	119.9
ACICU_00708	diguanylate cyclase	upstream mutation in -40 common t	intergenic mutation	24.6	19.7	13.8	21.5	26.0	27.9	17.2	26.5	23.8	19.5	18.9	15.9	305.4	230.8	213.0	198.4	342.6	11.9	11.2	20.7	14.8	19.0	19.6	23.8
ACICU_00728	hypothetical protein	none ID		113.3	83.7	88.4	45.1	59.7	76.4	94.6	61.8	152.0	427.4	472.0	591.5	283.7	187.0	340.6	229.1	104.3	1100.0	963.7	1092.9	1174.5	1632.3	1311.0	1218.5
ACICU_00823	leucyl/phenylalanyl-tRNA--protein transfera	ISAba1 upstream	insertion sequence	54.8	50.5	46.9	45.1	50.6	50.4	47.8	46.9	62.3	64.0	65.7	62.9	68.5	61.8	44.7	55.5	64.4	45.4	46.9	54.3	59.2	61.6	189.8	217.0
ACICU_00872	transcriptional regulator	sequence variation (adjacent to her	sequence variation	24.7	17.7	21.0	23.6	23.7	21.5	24.4	30.8	25.0	17.2	11.7	14.1	9.9	11.4	9.0	8.9	10.5	11.2	15.8	15.6	13.3	21.1	41.0	46.1
ACICU_01188	benzoate transporter	ISAba1 in ACICU_01189	insertion sequence	3.4	3.0	4.4	3.1	4.2	5.4	3.8	5.5	3.9	5.1	5.3	4.2	4.9	4.8	3.6	2.5	4.9	36.9	58.3	23.1	5.3	5.1	5.2	6.8
ACICU_01199	hypothetical protein	none ID		84.5	143.0	127.2	93.4	99.3	101.3	100.6	67.6	107.5	442.6	1070.4	905.6	190.7	195.2	57.8	147.4	63.0	108.7	371.3	314.3	329.8	249.7	1208.0	902.2
ACICU_01200	multidrug transporter MatE			67.1	66.7	67.7	45.4	58.0	55.3	82.1	48.4	94.7	73.9	91.0	81.8	157.8	78.5	43.1	77.0	52.1	85.8	67.8	73.1	88.9	62.3	14.3	15.5
ACICU_01262	type IV secretion protein Rhs	sequence variation	sequence variation	261.2	160.4	47.0	54.8	51.1	44.7	43.2	45.4	56.9	188.4	208.4	200.8	225.6	188.6	185.0	203.0	183.7	42.9	42.4	49.2	44.5	49.4	168.8	293.4
ACICU_01321	transcriptional regulator	ISAba1 very end of _01322 in P348.	insertion sequence	35.3	33.7	28.6	33.4	36.7	31.9	29.9	27.1	30.3	53.2	56.3	48.8	53.5	57.1	28.2	46.0	43.3	28.1	39.6	35.9	54.1	54.1	10.9	16.9
ACICU_01322	hemolysin			53.0	157.6	184.1	73.0	76.3	67.5	153.2	50.2	73.2	254.6	289.9	334.4	263.8	147.0	73.4	95.7	81.9	525.9	594.0	330.9	403.6	182.9	97.4	130.8
ACICU_01382	MFS transporter			41.6	24.8	32.8	27.9	27.5	23.0	44.8	33.8	45.2	32.9	29.6	24.4	4.0	3.5	2.4	2.9	2.8	1.5	3.7	2.2	2.4	3.8	2.4	3.7
ACICU_01383	acyl-CoA dehydrogenase	upstream mutation in -120 cladeA	intergenic mutation	99.5	82.9	93.8	77.4	73.1	56.6	101.7	73.3	112.8	112.7	92.4	77.0	6.3	4.8	4.0	4.3	3.6	2.8	3.5	4.6	6.9	4.6	3.8	6.7
ACICU_01474	hypothetical protein	clade C has ISAba1 between 73 an	insertion sequence	105.9	847.6	169.9	103.1	158.4	63.9	148.6	50.6	57.6	225.1	253.8	204.1	7.5	9.0	6.3	5.5	9.0	200.5	235.3	204.0	92.2	98.9	150.4	130.2
ACICU_01542	membrane protein	none ID		52.3	60.0	35.7	49.7	34.3	21.5	36.3	21.2	21.2	61.6	88.9	58.7	1364.0	1144.5	726.3	1002.3	715.2	40.7	23.0	53.2	20.5	17.9	27.9	22.4
ACICU_01560	blaOXA-51	ISAba1 variably upstream	insertion sequence	44.8	41.6	48.0	35.7	32.0	28.4	62.7	39.1	66.9	95.0	66.2	99.4	1189.3	1097.6	1035.2	1394.6	1326.2	66.1	132.3	60.4	1709.8	1591.6	1700.2	1436.5
ACICU_01720	ribonuclease D	ISAba12 in clade D, also ISAba125	insertion sequence	107.4	95.1	89.3	81.7	92.6	82.1	96.2	77.0	98.6	118.1	149.6	167.3	111.2	107.2	61.0	74.2	104.3	115.5	90.3	109.7	153.9	81.9	9.9	7.2
ACICU_01735	ABC transporter substrate-binding protein	clade BIS in ACICU_01735	insertion sequence	24.5	29.5	29.0	23.6	19.6	23.1	17.6	18.8	16.2	72.4	50.7	32.5	9.8	4.6	4.1	3.7	3.8	59.3	51.4	44.2	89.8	91.4	63.3	143.5
ACICU_01736	pyruvate dehydrogenase			53.9	41.9	45.0	40.4	40.1	46.5	43.7	30.0	31.3	113.7	72.7	64.5	29.2	23.5	16.9	18.9	16.7	84.6	60.2	77.8	125.4	137.5	112.2	169.3
ACICU_01737	diaminohydroxyphosphoribosylaminopyrimidine deaminase			28.2	27.0	26.5	22.5	21.9	17.7	19.7	19.3	16.6	49.5	35.3	27.5	17.6	16.2	10.2	11.3	10.2	42.8	37.6	34.4	52.6	57.3	59.3	79.1
ACICU_01738	dihydrolipoamide dehydrogenase			17.2	17.6	22.9	18.4	15.7	15.9	15.1	19.9	9.4	32.0	25.0	23.4	13.4	9.8	9.3	12.2	7.0	27.5	24.8	25.9	35.0	48.2	36.0	50.9
ACICU_01773	transporter	ISAba1 up from 01775 transF	insertion sequence	35.4	54.4	48.5	43.5	40.7	36.4	40.4	14.7	25.1	44.0	98.2	97.8	46.3	55.1	54.2	39.1	24.5	70.5	98.8	93.2	45.2	44.5	215.5	360.2
ACICU_01774	3-hydroxybutyrate dehydrogenase	ISAba1 IG, clade A,B,C	insertion sequence	17.3	17.1	19.5	12.2	12.5	15.8	12.6	9.6	14.1	48.9	66.9	53.0	23.6	24.4	27.1	13.1	10.3	47.3	56.3	68.7	45.4	62.8	146.2	229.2
ACICU_01781	phenol degradation protein meta	ISAba1 in ACICU_01780	insertion sequence	2.4	1.5	1.3	2.3	3.4	1.8	0.8	4.5	2.4	1.7	1.9	1.5	115.8	123.0	91.6	112.0	197.6	1.5	1.2	1.3	4.8	3.8	2.2	3.4
ACICU_01805	hypothetical protein	none ID		10.2	28.6	20.4	5.0	5.0	3.0	15.5	0.0	12.4	45.0	75.7	96.5	69.3	31.1	16.6	18.3	7.4	82.8	219.0	63.3	220.2	61.0	24.0	33.9
ACICU_01808	S-(hydroxymethyl)glutathione dehydrogenase			66.7	52.5	66.7	82.0	90.3	117.2	72.5	116.4	107.8	107.1	82.7	90.6	91.9	73.8	60.0	62.8	79.4	83.7	87.1	79.7	102.4	102.0	1815.0	1463.0
ACICU_01809	hypothetical protein			26.9	18.1	14.1	20.8	25.9	15.2	15.4	11.0	20.6	31.8	25.2	25.8	23.6	23.3	12.6	14.5	20.5	14.7	16.3	18.7	27.5	20.4	68.0	78.9
ACICU_01812	Type 1 P-pili pilus assembly protein	ISAba1 ACICU_01812, pilus	insertion sequence	64.7	53.5	45.2	50.2	61.9	45.1	51.9	38.8	50.1	147.6	79.3	65.0	78.8	90.8	54.6	81.3	115.8	40.9	62.5	91.2	74.0	60.1	18.7	25.8
ACICU_01920	antibiotic biosynthesis monooxygenase			438.8	342.7	313.3	306.1	229.3	171.8	244.2	199.5	459.9	260.9	427.8	442.2	475.4	515.4	492.5	548.4	232.2	242.7	183.0	115.8	4.4	4.7	1.4	2.1
ACICU_01921	TetR family transcriptional regulator	ISAba1 in 588	insertion sequence	20.7	15.4	15.6	23.6	16.1	19.3	21.7	20.4	11.0	29.3	22.8	21.9	16.1	22.5	14.5	15.8	21.8	11.4	21.5	16.9	260.3	197.4	0.2	0.3
ACICU_02067	hypothetical protein	none ID		42.2	51.8	49.6	25.8	32.7	15.3	28.0	18.0	35.7	107.0	103.4	153.7	26.2	54.4	7.9	15.8	14.0	148.6	173.1	89.7	133.7	81.7	116.6	52.4
ACICU_02107	amino acid dehydrogenase	ISAba1 upstream in clade A	insertion sequence	6.1	7.2	6.5	6.5	7.0	7.9	6.2	6.2	6.4	13.0	11.9	9.3	436.7	325.1	321.4	382.4	173.3	246.4	185.6	194.8	417.6	620.5	500.7	501.1
ACICU_02269	DNA breaking-rejoining protein	ISAba1, ISAba12	insertion sequence	12.3	27.7	21.7	1.8	2.6	2.2	18.3	0.3	7.2	39.3	68.9	140.2	43.0	16.1	6.9	15.4	4.4	67.9	154.9	100.4	160.6	74.7	147.4	144.8
ACICU_02270	hypothetical protein			626.3	1874.1	1178.0	226.4	254.8	217.7	988.7	165.0	586.9	5923.7	3733.5	5335.6	200.1	204.5	78.2	125.0	37.5	3330.8	8192.3	3272.0	5681.5	4246.2	3191.7	4294.1
ACICU_02361	transporter			21.8	20.9	24.2	14.9	19.5	15.2	17.7	15.5	14.6	21.6	30.7	25.8	26.3	26.1	12.5	21.1	23.7	18.4	22.2	18.7	32.5	24.9	87.7	87.2
ACICU_02362	N-glycosyltransferase	clade D ISAba1 in ACICU_02365	insertion sequence	10.2	7.0	6.8	7.4	8.8	6.0	8.0	5.2	5.6	11.6	12.3	11.5	13.3	9.0	7.9	5.4	8.0	9.4	9.3	9.1	9.0	9.7	64.8	65.6
ACICU_02363	pga transporter			11.3	8.2	9.6	7.8	7.9	7.7	9.5	3.7	7.0	13.4	12.8	12.0	11.6	10.8	7.0	8.4	11.1	9.5	8.3	11.0	10.0	12.5	66.0	57.5
ACICU_02365	biofilm synthesis protein			9.7	6.5	6.6	6.1	6.8	3.3	7.3	2.5	5.9	6.5	13.0	16.5	13.8	5.7	6.9	4.2	8.9	7.4	8.0	11.4	10.7	8.4	66.7	67.3
ACICU_02655	aromatic amino acid transporter	none ID		18.9	12.1	17.7	12.0	8.9	14.3	11.0	32.5	25.8	37.3	38.1	41.5	56.5	64.5	31.1	28.7	32.9	2.8	28.3	136.1	91.6	120.6	63.3	126.4
ACICU_02656	pyruvate decarboxylase			372.8	294.1	255.6	239.6	183.4	290.4	221.5	896.1	523.1	716.9	614.4	799.0	1528.5	1469.5	605.2	583.5	699.4	19.6	603.3	49.6	1965.1	1830.3	1119.5	1346.5
ACICU_02657	AsnC family transcriptional regulator			99.9	66.3	77.5	77.8	61.8	62.1	68.0	70.3	77.6	80.3	59.1	41.2	91.4	95.5	69.6	66.2	89.1	11.8	56.9	16.1	67.5	63.4	71.8	121.0
ACICU_02658	aldehyde dehydrogenase			999.1	839.0	826.6	754.0	726.6	930.3	765.5	1122.4	1219.2	1584.9	1558.3	1676.0	2165.6	1945.5	1333.6	1396.5	1188.6	193.9	1104.8	289.9	2010.8	2133.2	1483.8	2081.5
ACICU_02790	oxidoreductase	clade D ISAba1 in ACICU_02790	insertion sequence	89.5	63.5	51.8	71.7	90.3	79.5	59.0	61.0	78.7	111.1	85.8	102.6	85.1	66.3	63.7	84.4	74.1	53.7	58.9	67.4	62.5	70.8	14.7	17.6
ACICU_02938	type I secretion protein	ACICU_02946 ISAba27 upstream ir	insertion sequence	278.5	221.4	243.4	191.3	226.6	142.8	251.9	91.5	267.6	196.0	243.4	229.2	463.8	284.5	962.8	728.7	311.0	671.6	388.9	670.2	47.8	61.9	800.4	979.5
ACICU_02986	phosphatidylglycerophosphatase	none ID		287.1	268.9	304.1	334.8	312.4	406.3	262.3	273.4	287.6	364.1	329.5	216.5	544.5	622.2	630.7	579.8	556.3	92.6	88.1	150.7	97.6	108.2	391.9	523.0
ACICU_02987	hemolysin D			1362.8	1498.8	1318.5	1461.1	1422.3	1601.0	1460.0	1050.0	1509.8	2035.3	1863.7	1898.7	2080.6	2189.7	2341.3	2062.4	2353.6	705.3	640.0	826.6	737.3	685.1	2665.9	2097.7
ACICU_03330	succinylglutamate desuccinylase	sequence variation	sequence variation	56.9	59.1	56.3	55.8	45.9	63.8	59.2	76.0	89.3	42.7	72.5	86.7	84.2	75.9	100.4	115.5	59.6	63.5	88.9	82.8	113.0	123.5	221.3	286.7
ACICU_03331	N-succinylarginine dihydrolase	arginine to gluatmate metabolism		215.8	150.9	125.3	157.7	170.1	189.4	194.3	174.0	309.8	145.2	135.4	197.6	246.8	233.6	294.2	328.3	180.3	113.6	135.4	182.8	206.6	332.2	560.0	729.1
ACICU_03332	succinylglutamate-semialdehyde dehydrogenase			169.5	143.4	129.7	151.5	145.2	206.0	181.0	157.2	265.9	135.2	172.6	190.7	254.6	225.5	236.4	274.7	167.0	155.3	129.1	203.0	192.0	330.4	470.8	668.4
ACICU_03333	arginine N-succinyltransferase			199.9	161.1	148.6	175.6	164.6	224.5	197.1	158.4	230.0	142.9	248.3	270.0	262.9	249.0	202.7	184.9	241.4	222.1	187.7	320.2	183.0	360.7	577.2	735.3
ACICU_03391	pilus assembly protein PilP	sequence divergence between clad	sequence variation	5.9	3.2	5.0	11.9	11.8	13.4	5.0	13.5	4.5	1.9	6.1	2.5	2.8	2.4	1.5	4.4	6.6	1.4	1.4	2.8	2.4	3.4	1.4	2.0
ACICU_03394	pilus assembly protein PilM			224.0	80.3	82.0	304.2	270.2	347.0	146.1	245.8	186.6	158.2	93.2	85.8	98.3	126.3	102.4	104.7	167.3	35.9	26.9	49.9	44.2	67.7	66.0	57.2
ACICU_03432	hypothetical protein, clade D identical to AC	sequence divergence	sequence variation	322.3	151.9	89.5	303.6	349.8	338.1	143.8	160.8	290.1	116.6	123.6	135.8	250.4	200.9	227.6	292.8	265.4	134.5	75.4	207.0	175.9	199.7	71.4	87.8
ACICU_03436	transporter			10.3	9.4	7.6	10.7	12.5	15.7	10.4	11.8	9.3	16.6	9.7	9.1	16.7	9.9	8.4	9.8	11.8	6.5	6.0	11.0	12.0	13.2	41.4	19.3
ACICU_03437	membrane protein			82.9	66.3	45.6	69.4	65.6	74.2	50.3	59.4	96.8	48.6	50.6	49.1	23.4	16.0	11.3	13.7	21.2	54.7	50.0	74.2	79.2	87.2	103.4	113.3
ACICU_03438	cold-shock protein			11.1	12.8	8.4	5.7	10.1	9.0	5.9	10.9	12.5	13.9	17.0	17.7	41.2	74.5	51.4	64.3	54.3	13.1	10.3	13.2	15.6	12.8	8.4	8.0
ACICU_03471	methyltransferase			36.5	38.3	33.5	25.6	29.7	29.4	25.5	49.8	52.2	33.4	34.1	48.1	39.2	45.6	35.4	35.9	47.3	26.9	18.8	30.5	50.1	47.8	6.8	7.8
ACICU_03472	monoamine oxidase	sequence variation, also ISAba125 i	sequence variation	181.9	182.3	148.6	149.7	146.6	196.1	136.4	223.5	277.5	191.2	167.0	224.0	303.5	277.6	197.4	207.2	285.2	139.5	244.6	140.7	221.9	251.6	30.7	40.8
ACICU_03575	lipase	sequence variation	sequence variation	12.0	13.2	10.3	12.8	13.4	10.9	9.1	4.3	14.5	14.7	16.4	16.3	20.3	13.4	13.9	15.0	13.5	13.0	11.9	14.8	15.3	10.5	59.5	117.0
ACICU_03618	nicotinamidase	sequence variation	sequence variation	69.9	57.6	49.4	70.1	67.8	87.4	68.2	69.2	71.3	102.9	89.3	77.8	86.3	76.3	54.6	72.8	89.5	55.7	57.4	56.8	101.7	91.0	373.7	232.6
ACICU_03627	membrane protein	none ID		232.5	454.6	578.2	133.2	116.3	74.1	425.9	50.5	175.1	594.5	538.6	516.6	817.3	759.8	255.0	434.7	211.4	1079.9	1885.4	667.8	1073.5	536.5	133.7	292.4
		intergenic	3																								
		insertion sequence	19																								
		sequence variation	8																								
