# Supplementary material for: Transcriptome Remodeling of Acinetobacter baumannii during Infection and Treatment
Source: mBio. 2017 Mar 7;8(2):e02193-16. doi: 10.1128/mBio.02193-16 (PMC5340874; doi:10.1128/mBio.02193-16)
Supplement: TABLE S5 [file mbo001173221st5.pdf]

Table S5. The most variably expressed core genes among isolates

				Clade	A												B					C					D	
Locus Tag	also variable among clades?	predicted annotation	specific gene event	type of change	ABUH315100	ABUH315101	ABUH28081	ABUH28092	ABUH28093	ABUH28099	ABUH475197	ABUH475239	ABUH475361	ABUH81366	ABUH81389	ABUH81452	ABUH66241	ABUH66253	ABUH66268	ABUH66271	ABUH66276	ABUH41096	ABUH410103	ABUH410128	ABUH588656	ABUH588663	ABUH34813	ABUH34827
ACICU_00072		wzb, protein tyrosine phosphatase	sequence variation	sequence variation	611.0	560.7	483.6	439.7	582.6	494.5	552.8	405.3	676.9	913.7	1060.9	1301.5	653.1	718.5	549.7	723.0	393.2	1228.6	1292.4	1082.3	19.3	9.2	73.2	88.8
ACICU_00087		UDP-galactose phosphate transferase	ISAba125 between ACICU_00086-87 in 66276	insertion sequence	379.1	335.6	327.0	385.7	397.4	331.2	336.6	309.2	476.9	319.6	282.8	296.8	415.0	343.7	301.9	355.6	27.8	327.3	331.0	294.6	777.3	649.6	4.2	3.9
ACICU_00093		L-lactate permease	None ID	none identified	205.9	161.3	194.0	156.6	197.7	191.0	165.8	101.7	204.2	119.2	263.2	208.3	122.6	52.8	191.5	138.2	118.8	151.3	7.7	128.8	14.6	18.5	771.3	22.4
ACICU_00094		hypothetical protein			62.0	42.2	67.8	42.2	68.2	48.6	50.9	49.7	59.0	43.5	64.4	47.7	23.9	23.0	55.8	39.2	26.4	51.1	8.2	35.1	62.5	106.9	422.5	27.9
ACICU_00095		lactate dehydrogenase			285.8	167.5	177.2	140.4	215.1	237.6	178.2	203.7	333.2	176.1	326.6	287.0	219.5	89.7	306.1	214.1	160.1	196.3	21.3	173.9	41.2	68.5	649.7	43.5
ACICU_00278		RND transporter	None ID	none identified	18.7	14.5	14.1	11.4	14.0	16.9	13.2	30.2	23.2	68.2	59.4	76.8	22.7	18.9	21.7	18.5	11.6	47.7	61.0	70.6	88.0	84.7	74.9	59.2
ACICU_00290		Type II general secretion pathway protein	None ID	none identified	26.0	10.7	10.1	12.6	11.7	19.4	12.6	31.5	23.1	67.3	60.9	74.0	14.7	17.0	26.2	15.3	15.9	79.7	51.8	87.6	68.6	97.2	89.2	86.3
ACICU_00306	Y	hypothetical protein	ISAba1 in middle of 00307, P410 and P588	insertion sequence	1.5	0.7	1.6	1.3	0.0	0.0	0.4	1.7	2.2	5.8	10.4	7.0	3.0	1.2	5.8	1.7	1.2	8.9	7.6	12.4	19.7	41.9	17.2	21.5
ACICU_00307	Y	hot-dog fold hypothetical protein			47.8	62.0	35.2	48.4	49.1	52.4	40.4	26.8	43.1	84.2	73.7	67.7	45.1	47.9	50.0	73.5	39.4	420.2	392.5	468.1	463.2	415.7	52.1	48.0
ACICU_00318		signal peptide-containing protein	None ID	none identified	14.0	30.0	19.6	7.8	9.3	15.2	24.1	7.0	17.5	81.3	60.2	74.2	80.9	39.1	35.2	38.5	21.4	113.5	390.1	90.0	202.1	78.6	40.1	37.3
ACICU_00450	Y	PF03729 repeat protein	None ID		44.8	67.0	44.3	31.9	27.2	29.3	37.5	25.2	28.0	67.6	156.4	187.0	216.9	70.5	58.5	82.8	120.9	82.0	109.5	183.3	192.4	233.8	342.7	356.4
ACICU_00601		50S ribosomal protein L20	None ID		4900.1	4407.9	5056.6	5532.0	3446.9	4514.3	3663.8	4711.4	3678.9	1682.1	1752.1	1688.5	2239.7	3906.2	4562.9	4088.6	3632.4	1672.2	1446.1	2024.5	1111.8	947.6	1141.2	1833.2
ACICU_00621		organic hydroperoxide resistance protein	TtoG at -46 between transR and this gene	intergenic mutation	25.7	57.1	75.5	34.5	27.5	28.4	33.4	16.9	36.6	98.1	85.2	80.8	38.4	40.3	44.3	33.9	40.0	398.0	399.6	386.3	216.6	199.1	80.0	52.4
ACICU_00684	Y	membrane protein	clade C has G to A @761268, start is at 761264, ACICU has upstrean	intergenic mutation	26.4	32.2	19.3	6.6	7.3	6.4	19.7	10.5	95.0	299.5	242.3	327.8	113.1	68.6	62.9	45.1	8.2	707.4	1250.5	351.6	421.5	931.1	147.4	119.9
ACICU_00708	Y	diguanylate cyclase	upstream mutation in -40 common to clade B	intergenic mutation	24.6	19.7	13.8	21.5	26.0	27.9	17.2	26.5	23.8	19.5	18.9	15.9	305.4	230.8	213.0	198.4	342.6	11.9	11.2	20.7	14.8	19.0	19.6	23.8
ACICU_00728	Y	hypothetical protein	None ID	none identified	113.3	83.7	88.4	45.1	59.7	76.4	94.6	61.8	152.0	427.4	472.0	591.5	283.7	187.0	340.6	229.1	104.3	1100.0	963.7	1092.9	1174.5	1632.3	1311.0	1218.5
ACICU_00761		trehalose-6-phosphate synthase	None ID	none identified	166.1	371.4	270.6	34.1	33.4	20.5	354.5	16.4	314.0	675.2	534.2	981.1	1821.6	835.2	537.1	595.6	133.6	1173.9	3097.2	1152.9	1518.0	923.0	568.8	445.5
ACICU_00762		trehalose phosphatase			9.2	48.6	37.9	4.5	3.6	1.6	28.3	0.8	16.7	18.1	35.8	84.5	180.0	81.8	22.0	35.6	7.4	134.6	537.4	128.0	174.9	60.7	36.9	35.7
ACICU_00839		acetylglutamate kinase	None ID	none identified	88.0	54.1	66.1	34.4	38.4	47.5	39.9	99.1	84.4	191.6	238.0	256.1	131.5	99.4	133.7	78.1	49.8	297.0	278.3	248.2	239.9	353.3	276.4	289.1
ACICU_00881		hypothetical protein	sequence variation, associated with hemO recombinant region	sequence variation	27.5	17.0	8.1	12.9	12.1	11.4	15.6	19.3	22.3	1.2	1.0	1.2	1.5	0.5	1.8	2.3	1.0	11.5	15.8	8.5	5.1	3.6	2.4	1.1
ACICU_00888		choline dehydrogenase		sequence variation	818.4	898.7	1146.8	1058.2	1333.5	446.3	1414.8	145.3	333.0	293.4	286.7	145.4	153.4	149.6	324.0	194.1	770.0	333.1	143.4	102.5	77.2	113.0	113.6	60.0
ACICU_00889		betaine-aldehyde dehydrogenase			1203.1	1437.8	1750.7	1560.7	1963.9	801.6	1992.4	310.9	665.6	473.2	402.8	200.2	245.2	274.7	412.7	312.0	737.9	511.0	251.9	199.1	133.0	213.1	196.0	157.5
ACICU_00890		BetI family transcriptional regulator			613.5	869.4	1307.0	1180.7	858.1	614.2	919.9	250.3	368.4	699.0	643.4	325.2	504.3	595.1	758.8	603.9	1418.5	313.4	210.2	106.8	85.3	110.4	178.6	186.7
ACICU_00891		choline transporter			206.5	219.4	298.6	265.2	267.7	128.5	274.6	57.8	112.4	130.8	124.5	74.9	73.7	95.2	100.9	68.0	215.4	68.7	56.3	32.9	29.3	55.5	77.1	38.4
ACICU_00895		hypothetical protein			21.9	152.0	224.2	23.9	11.6	14.8	170.1	77.5	346.8	83.1	123.4	125.0	347.4	218.7	79.3	88.4	121.9	277.6	525.0	254.9	542.6	567.4	68.8	70.0
ACICU_01023		hypothetical protein	phage region, variable sequence	sequence variation	123.5	95.6	109.5	139.2	120.4	103.5	93.7	66.6	0.0	252.4	212.0	236.5	83.4	129.1	95.4	112.4	95.0	209.8	203.8	179.7	503.3	486.5	628.5	907.5
ACICU_01027		hypothetical protein			55.8	107.1	64.3	25.3	20.2	18.7	56.6	11.2	0.0	183.0	347.0	856.6	314.8	119.5	40.6	106.0	37.0	307.4	1803.5	385.4	2037.9	873.9	410.3	337.0
ACICU_01072		lipid A phosphoethanolamine transferase	pmr related	sequence variation	17.9	15.2	15.6	39.4	51.6	254.5	11.9	4.4	11.2	14.4	21.1	27.3	13.2	80.5	8.3	10.5	13.1	13.6	15.3	16.2	10.7	137.0	111.4	219.2
ACICU_01172		outer membrane protein	ISAba1 up from ACICU_01172 in P588	insertion sequence	200.4	133.8	95.2	122.3	106.6	106.7	99.8	111.1	523.2	208.5	204.8	263.2	451.5	489.6	339.9	325.6	140.9	268.1	619.7	322.8	16.4	13.5	332.9	236.2
ACICU_01188	Y	benzoate transporter	ISAba1 in middle of 01189, expression up of adjacent 01188, benzoate	insertion sequence	3.4	3.0	4.4	3.1	4.2	5.4	3.8	5.5	3.9	5.1	5.3	4.2	4.9	4.8	3.6	2.5	4.9	36.9	58.3	23.1	5.3	5.1	5.2	6.8
ACICU_01194		heavy metal transporter	None ID	none identified	159.5	381.0	263.2	241.9	321.8	194.0	306.5	134.8	101.8	45.5	53.4	45.2	84.1	121.5	97.5	131.4	193.2	50.9	86.6	43.2	97.8	51.5	55.3	75.3
ACICU_01195		ATPase			104.6	170.0	74.0	112.6	193.6	112.7	165.0	61.9	57.0	30.7	27.6	24.8	54.2	52.9	80.9	111.4	104.7	17.2	21.8	32.0	27.5	32.1	31.9	30.9
ACICU_01198		cysteine-rich helical bundle repeat protein			2035.9	2994.7	1948.1	741.5	789.5	583.9	3001.1	535.8	2178.8	7270.0	4783.8	7899.0	12822.5	4866.3	4342.9	5843.4	1310.7	4063.5	18493.2	4739.1	18920.8	9407.1	5518.6	4352.8
ACICU_01199	Y	hypothetical protein			84.5	143.0	127.2	93.4	99.3	101.3	100.6	67.6	107.5	442.6	1070.4	905.6	190.7	195.2	57.8	147.4	63.0	108.7	371.3	314.3	329.8	249.7	1208.0	902.2
ACICU_01261		type VI secretion system protein	sequence variation	sequence variation	72.0	56.7	37.6	37.2	43.7	35.3	38.0	21.1	48.2	120.0	130.1	139.0	84.2	79.6	68.9	81.8	53.5	122.4	97.0	114.0	49.4	53.0	105.3	172.8
ACICU_01262	Y	type VI secretion protein Rhs			261.2	160.4	47.0	54.8	51.1	44.7	43.2	45.4	56.9	188.4	208.4	200.8	225.6	188.6	185.0	203.0	183.7	42.9	42.4	49.2	44.5	49.4	168.8	293.4
ACICU_01322	Y	hemolysin	ISAba1 very end of _01322 in P348,ISAba125 in 475239 in 1321	insertion sequence	53.0	157.6	184.1	73.0	76.3	67.5	153.2	50.2	73.2	254.6	289.9	334.4	263.8	147.0	73.4	95.7	81.9	525.9	594.0	330.9	403.6	182.9	97.4	130.8
ACICU_01335		enoyl-CoA hydratase	sequence variation	sequence variation	323.0	403.4	464.1	370.0	290.0	347.9	305.5	422.0	303.3	484.1	565.2	540.5	734.6	709.6	509.9	396.5	442.1	20.1	464.4	37.7	826.2	479.8	1003.5	1073.0
ACICU_01336		ATPase AAA			1870.5	2064.1	2332.3	2065.1	1489.4	1698.3	1543.7	2145.3	1656.9	2995.2	3693.6	3126.0	4555.6	5140.5	3139.1	2648.7	2387.5	73.9	2758.8	187.5	4643.8	3031.5	4842.0	5116.8
ACICU_01337		phenylacetate-CoA oxygenase			237.7	420.4	482.4	293.0	183.2	297.9	210.3	461.7	241.7	390.1	565.7	382.8	736.5	888.8	406.9	293.6	403.8	16.2	552.3	34.7	998.4	377.1	744.1	922.6
ACICU_01338		phenylacetic acid degradation protein			756.6	1217.0	1428.5	852.8	696.8	923.6	828.5	1728.1	895.7	1242.4	1770.0	1649.0	2397.6	3170.6	1212.2	1038.1	1133.4	47.2	1677.9	94.3	2770.4	1727.5	2941.4	2806.6
ACICU_01339		phenylacetate-CoA oxygenase			1454.4	1941.8	2048.6	1293.1	1085.2	1453.7	1418.9	2408.3	1330.1	1680.7	2619.5	2664.0	3963.7	3776.1	2425.1	2039.2	2218.1	56.2	2245.2	142.7	4009.9	2764.9	5125.0	5454.5
ACICU_01340		phenylacetic acid degradation protein			379.1	427.2	459.4	287.0	274.9	366.4	298.6	756.1	448.7	466.2	671.6	735.2	1074.9	1027.4	640.2	516.4	521.0	20.4	580.4	35.1	1195.1	983.2	1393.6	1698.3
ACICU_01341		2,3-dehydroadipyl-CoA hydratase			107.4	98.6	111.7	58.5	58.0	101.5	71.5	213.8	109.1	143.9	254.4	227.6	311.3	261.5	214.2	146.4	149.7	8.6	177.2	11.5	423.5	377.3	461.6	610.3
ACICU_01342		enoyl-CoA hydratase			478.2	338.9	446.9	226.1	237.6	414.9	308.1	911.2	435.2	483.7	757.8	728.5	1344.7	1101.4	1018.0	611.3	604.0	25.9	305.3	34.8	1102.2	1328.1	1405.7	1689.1
ACICU_01343		3-hydroxyacyl-CoA dehydrogenase			376.8	326.0	423.5	221.6	204.2	306.7	272.7	609.5	301.0	372.1	539.0	539.9	1081.6	977.6	727.7	532.3	514.3	13.2	247.0	24.3	987.9	853.0	971.1	981.9
ACICU_01344		beta-ketoadipyl CoA thiolase			250.1	223.9	295.7	159.5	129.6	195.0	177.2	449.2	220.4	306.2	357.6	351.6	803.2	663.1	522.0	331.5	340.6	10.9	140.4	17.1	689.0	532.8	600.5	766.9
ACICU_01345		phenylacetate--CoA ligase			88.0	66.8	103.9	45.3	38.0	58.3	44.9	276.7	84.2	141.6	245.0	237.4	217.2	182.1	229.9	88.0	117.9	19.1	115.5	21.8	392.0	396.7	488.8	783.7
ACICU_01382	Y	MFS transporter	upstream mutation in -120 cladeA	intergenic mutation	41.6	24.8	32.8	27.9	27.5	23.0	44.8	33.8	45.2	32.9	29.6	24.4	4.0	3.5	2.4	2.9	2.8	1.5	3.7	2.2	2.4	3.8	2.4	3.7
ACICU_01383	Y	acyl-CoA dehydrogenase			99.5	82.9	93.8	77.4	73.1	56.6	101.7	73.3	112.8	112.7	92.4	77.0	6.3	4.8	4.0	4.3	3.6	2.8	3.5	4.6	6.9	4.6	3.8	6.7
ACICU_01419		hypothetical protein	None ID	none identified	2.7	9.7	27.6	3.5	1.2	0.0	9.3	0.0	3.0	16.2	26.3	14.4	27.0	13.1	3.5	3.2	4.6	26.5	114.0	6.3	30.4	3.2	6.2	6.4
ACICU_01425		hypothetical protein	is variation in ACICU_01429 though		433.7	673.3	557.7	139.0	124.0	46.3	618.8	23.1	266.7	610.7	841.5	1360.2	1443.5	556.6	514.0	795.9	221.0	987.1	2402.1	1065.7	1532.2	761.4	487.3	758.6
ACICU_01426		hydroperoxidase			1399.8	1792.1	1305.1	319.9	397.2	227.2	1608.4	64.2	1217.8	2144.5	2515.4	3218.1	4544.7	2303.3	2068.7	2631.4	961.0	3102.9	4390.2	2449.5	4324.3	2234.8	2000.8	3194.7
ACICU_01472		ubiquinol oxidase subunit II	101 has mut in lysR, ACICU_01470	mutation	34.0	752.5	42.9	29.3	26.9	37.0	42.0	29.9	28.8	92.8	104.0	127.8	193.2	142.6	184.6	164.4	190.7	157.4	338.2	136.9	170.4	165.5	99.1	103.4
ACICU_01473		cytochrome D ubiquinol oxidase subunit I			23.5	714.1	44.4	23.2	21.6	27.4	35.2	25.6	25.6	83.4	103.9	125.8	159.3	122.0	135.3	137.0	177.8	166.6	324.5	158.1	174.4	145.9	94.2	89.0
ACICU_01474	Y	hypothetical protein	clade C has ISAba1 between ACICU_01473 and 74	insertion sequence	105.9	847.6	169.9	103.1	158.4	63.9	148.6	50.6	57.6	225.1	253.8	204.1	7.5	9.0	6.3	5.5	9.0	200.5	235.3	204.0	92.2	98.9	150.4	130.2
ACICU_01518		lipoprotein	pmr related	mutation	58.0	61.0	301.3	498.8	682.4	2679.0	76.8	36.6	92.7	100.2	707.8	688.0	134.8	4086.9	77.2	93.3	52.1	71.3	110.0	66.6	88.4	7766.2	1513.3	6770.4
ACICU_01542	Y	membrane protein	None ID	none identified	52.3	60.0	35.7	49.7	34.3	21.5	36.3	21.2	21.2	61.6	88.9	58.7	1364.0	1144.5	726.3	1002.3	715.2	40.7	23.0	53.2	20.5	17.9	27.9	22.4
ACICU_01552		hypothetical protein	pmr related	mutation	14.0	12.4	49.8	419.7	429.0	2374.3	9.3	4.7	14.8	17.6	208.7	304.6	16.1	1493.0	2.8	8.4	10.4	8.0	23.7	19.5	12.6	4688.1	269.0	1824.3
ACICU_01553		hypothetical protein			18.5	19.7	29.2	56.7	45.2	252.1	18.7	16.2	21.8	16.1	49.2	46.9	11.6	168.9	10.9	16.8	17.6	22.1	33.4	25.6	25.1	402.1	44.6	232.8
ACICU_01560	Y	blaOXA51	blaoxa51-like with/without ISAba1	insertion sequence	44.8	41.6	48.0	35.7	32.0	28.4	62.7	39.1	66.9	95.0	66.2	99.4	1189.3	1097.6	1035.2	1394.6	1326.2	66.1	132.3	60.4	1709.8	1591.6	1700.2	1436.5
ACICU_01672		siderophore biosynthesis protein	duplication in ABUH41096	duplication	15.7	11.5	11.2	13.2	11.9	7.7	11.4	10.4	14.9	15.3	24.3	20.4	22.0	14.2	11.0	9.9	11.0	14.0	10.5	12.5	21.9	14.9	355.6	13.0
ACICU_01673		ornithine monooxygenase	feoB mutation in ABUH34813	mutation	7.7	7.2	7.7	7.4	9.2	3.4	9.0	8.8	7.3	10.0	13.0	11.0	8.8	7.4	5.3	6.5	6.8	36.7	8.4	6.6	16.5	9.7	184.3	6.8
ACICU_01679		ligand-gated channel protein			145.3	66.5	83.7	119.7	138.7	78.2	129.7	96.4	127.0	73.9	65.4	55.1	111.0	70.0	77.0	66.5	95.4	595.9	52.4	61.7	79.2	60.9	1484.5	298.8
ACICU_01683		siderophore biosynthesis protein			60.8	31.9	46.1	52.8	54.3	40.7	56.0	75.1	78.8	85.5	51.6	49.8	72.5	52.1	43.6	39.9	51.3	1904.9	46.0	39.0	86.0	57.5	784.4	123.3
ACICU_01720	Y	ribonuclease D	ISAba12 in clade D	insertion sequence	107.4	95.1	89.3	81.7	92.6	82.1	96.2	77.0	98.6	118.1	149.6	167.3	111.2	107.2	61.0	74.2	104.3	115.5	90.3	109.7	153.9	81.9	9.9	7.2
ACICU_01727		von Willebrand factor A	missing ACICU_01721-26 in ABUH34827	deletion	335.8	293.6	330.4	317.2	353.0	289.8	304.7	284.6	331.7	340.0	552.7	440.3	376.3	351.4	250.4	280.6	271.9	322.7	228.4	344.7	262.0	270.9	284.3	12.0
ACICU_01735	Y	ABC transporter substrate-binding protein	IS in ACICU_01735 clade B	insertion sequence	24.5	29.5	29.0	23.6	19.6	23.1	17.6	18.8	16.2	72.4	50.7	32.5	9.8	4.6	4.1	3.7	3.8	59.3	51.4	44.2	89.8	91.4	63.3	143.5
ACICU_01740		butanediol dehydrogenase	clade D ISAba1 in ACICU_01739	insertion sequence	15.1	11.4	13.8	12.6	11.7	15.0	10.3	13.5	15.4	21.9	20.0	21.7	16.1	20.1	15.3	12.8	12.8	23.0	25.8	22.8	48.4	44.9	734.8	773.1
ACICU_01762		aspartate ammonia-lyase	PS ABUH66276 ISAba1 in ACICU_01765	insertion sequence	903.8	1204.4	879.0	777.8	913.6	674.9	745.2	1211.6	722.1	1614.0	1587.1	1864.2	1426.8	928.1	710.4	672.6	4.4	1004.2	885.8	1759.6	829.6	1073.0	582.3	305.8
ACICU_01774	Y	3-hydroxybutyrate dehydrogenase	ISAba1 IG, end of ACICU_01774, clade A,B,C	insertion sequence	17.3	17.1	19.5	12.2	12.5	15.8	12.6	9.6	14.1	48.9	66.9	53.0	23.6	24.4	27.1	13.1	10.3	47.3	56.3	68.7	45.4	62.8	146.2	229.2
ACICU_01781	Y	phenol degradation protein meta	clade B ISAba1 in ACICU_01780	insertion sequence	2.4	1.5	1.3	2.3	3.4	1.8	0.8	4.5	2.4	1.7	1.9	1.5	115.8	123.0	91.6	112.0	197.6	1.5	1.2	1.3	4.8	3.8	2.2	3.4
ACICU_01805	Y	hypothetical protein	None ID	none identified	10.2	28.6	20.4	5.0	5.0	3.0	15.5	0.0	12.4	45.0	75.7	96.5	69.3	31.1	16.6	18.3	7.4	82.8	219.0	63.3	220.2	61.0	24.0	33.9
ACICU_01808	Y	S-(hydroxymethyl)glutathione dehydrogenas	ISAba1 in clade A,B,C in ACICU_01812	insertion sequence	66.7	52.5	66.7	82.0	90.3	117.2	72.5	116.4	107.8	107.1	82.7	90.6	91.9	73.8	60.0	62.8	79.4	83.7	87.1	79.7	102.4	102.0	1815.0	1463.0
ACICU_01823		AdeC	adeRS mutations	mutation	56.9	63.6	4.4	6.1	4.7	4.9	6.3	4.5	3.9	60.5	9.2	7.0	26.0	529.6	43.5	38.1	168.2	51.6	27.9	47.5	113.3	115.5	42.5	45.5
ACICU_01824		AdeB			90.6	85.6	3.6	6.2	5.0	3.8	3.8	3.8	2.8	130.8	5.4	5.2	64.9	1039.1	69.2	74.7	235.7	92.1	36.8	87.6	194.1	220.1	92.3	100.8
ACICU_01825		AdeA	5bp deletion at -40 from adeA in ABUH81389 and ABUH81452	intergenic mutation	87.3	84.6	6.1	11.7	11.6	11.7	9.1	7.6	7.8	109.5	4.6	4.6	82.9	1286.9	76.8	78.6	224.3	119.0	34.2	89.5	120.8	147.7	82.4	94.6
ACICU_02067	Y	hypothetical protein	None ID	none identified	42.2	51.8	49.6	25.8	32.7	15.3	28.0	18.0	35.7	107.0	103.4	153.7	26.2	54.4	7.9	15.8	14.0	148.6	173.1	89.7	133.7	81.7	116.6	52.4
ACICU_02071		hypothetical protein			17.5	56.7	56.9	7.2	9.1	3.7	45.1	5.4	11.7	50.9	78.9	82.2	68.3	33.1	21.0	35.0	10.0	67.6	232.8	64.5	101.4	56.3	25.0	32.3
ACICU_02107	Y	amino acid dehydrogenase	ISAba1 up from ACICU_02107 in clade A	insertion sequence	6.1	7.2	6.5	6.5	7.0	7.9	6.2	6.2	6.4	13.0	11.9	9.3	436.7	325.1	321.4	382.4	173.3	246.4	185.6	194.8	417.6	620.5	500.7	501.1
ACICU_02262		magnesium ABC transporter ATPase	None ID	none identified	4.4	4.3	2.5	4.1	4.4	3.6	4.1	5.4	3.3	47.4	19.1	24.8	3.6	5.1	2.8	3.1	4.5	5.5	5.1	7.4	39.7	59.8	56.9	14.4
ACICU_02269	Y	DNA breaking-rejoining protein	see text, ISAba1 and ISAba12	insertion sequence	12.3	27.7	21.7	1.8	2.6	2.2	18.3	0.3	7.2	39.3	68.9	140.2	43.0	16.1	6.9	15.4	4.4	67.9	154.9	100.4	160.6	74.7	147.4	144.8
ACICU_02270	Y	hypothetical protein			626.3	1874.1	1178.0	226.4	254.8	217.7	988.7	165.0	586.9	5923.7	3733.5	5335.6	200.1	204.5	78.2	125.0	37.5	3330.8	8192.3	3272.0	5681.5	4246.2	3191.7	4294.1
ACICU_02289		hypothetical protein	None ID	none identified	57.7	93.2	77.4	44.1	44.5	57.2	90.4	27.9	72.4	318.7	179.3	229.8	397.5	281.1	64.1	89.1	104.6	138.2	601.8	210.3	361.9	319.0	168.0	122.3
ACICU_02347		AMP-binding protein	None ID	none identified	57.1	48.5	37.3	30.2	24.5	24.2	37.7	22.5	38.2	95.5	224.9	200.5	129.3	139.9	132.4	148.0	16.3	120.0	270.2	143.8	200.1	173.7	169.2	180.8
ACICU_02348		acyl-CoA dehydrogenase			41.0	34.7	27.8	18.5	19.1	18.5	17.8	15.1	38.6	54.6	170.5	168.6	80.4	76.7	90.7	81.9	11.3	97.1	143.3	101.2	107.4	119.8	93.0	122.6
ACICU_02349		3-hydroxy-2-methylbutyryl-CoA dehydrogenase			116.7	72.9	65.2	51.7	42.1	48.9	74.7	38.0	94.7	180.0	400.5	401.7	233.1	202.8	213.1	262.0	27.8	160.2	298.3	212.6	257.4	308.2	208.0	245.2
ACICU_02385		hypothetical protein	None ID	none identified	34.9	104.1	58.3	20.8	23.3	16.3	53.6	18.6	60.1	404.7	229.9	448.3	196.1	126.3	74.0	159.1	36.7	448.7	688.3	436.9	1138.8	390.7	614.6	292.0
ACICU_02431		DNA-binding protein	see text	insertion sequence	288.2	429.4	381.6	59.2	63.7	32.0	371.1	24.2	136.4	535.9	479.6	617.1	751.2	422.9	397.0	403.6	97.7	447.2	726.1	550.4	795.0	325.2	135.5	168.7
ACICU_02432		glycosyl transferase		deletion	54.7	72.0	35.0	8.1	13.8	12.9	53.1	5.2	48.3	249.3	223.7	324.2	262.8	102.0	105.0	166.3	36.3	334.5	522.7	276.0	643.9	316.9	231.5	207.3
ACICU_02433		methyltransferase			31.5	48.7	26.6	5.3	3.3	5.0	26.9	4.5	32.3	91.0	101.7	143.0	172.0	61.4	51.0	87.0	20.5	145.4	224.2	138.1	255.1	152.2	91.8	91.5
ACICU_02434		LmbE protein			34.0	52.7	31.2	7.4	5.0	8.5	27.1	3.5	33.9	68.0	107.7	182.0	220.9	74.7	89.7	116.5	20.1	161.0	248.6	138.8	229.7	171.4	98.8	116.4
ACICU_02435		acyl-CoA dehydrogenase			14.8	28.7	22.8	4.4	5.3	2.9	17.1	3.3	16.7	32.9	59.8	81.4	100.3	36.5	42.6	50.9	9.3	96.7	169.6	80.2	139.7	107.3	52.4	69.2
ACICU_02488		hypothetical protein	pmr related	mutation	141.1	159.5	181.2	218.6	228.0	353.8	134.6	61.6	166.9	1246.6	1264.7	1926.2	233.1	545.1	93.6	101.1	100.2	189.8	287.0	231.0	475.3	3186.2	1798.8	4266.3
ACICU_02566		hypothetical protein	None ID	none identified	1905.9	976.9	1085.0	1757.4	1445.6	1520.3	942.5	705.0	2259.4	451.5	645.4	525.9	1074.0	1020.5	1245.3	972.9	1559.8	509.3	280.5	901.9	428.1	365.5	248.1	374.0
ACICU_02655	Y	aromatic amino acid transporter	None ID	none identified	18.9	12.1	17.7	12.0	8.9	14.3	11.0	32.5	25.8	37.3	38.1	41.5	56.5	64.5	31.1	28.7	32.9	2.8	28.3	136.1	91.6	120.6	63.3	126.4
ACICU_02656	Y	pyruvate decarboxylase			372.8	294.1	255.6	239.6	183.4	290.4	221.5	896.1	523.1	716.9	614.4	799.0	1528.5	1469.5	605.2	583.5	699.4	19.6	603.3	49.6	1965.1	1830.3	1119.5	1346.5
ACICU_02783		hypothetical protein	None ID	none identified	39.4	24.9	30.0	23.1	23.4	35.5	20.1	69.5	41.0	181.7	147.3	124.7	33.9	37.3	53.0	31.1	41.6	90.7	136.4	105.0	132.1	194.1	159.9	191.4

ACICU_02866		glycosyl transferase family 2	pmr related	mutation	2.7	2.2	2.9	9.2	7.6	111.9	0.9	1.9	1.6	2.7	4.1	4.6	1.4	35.0	3.1	1.6	1.1	2.2	2.4	3.9	1.9	92.6	41.9	53.8
ACICU_02867		polysaccharide biosynthesis protein GtrA			1.9	1.7	0.5	2.7	4.5	44.5	0.0	0.8	0.5	1.5	2.0	2.2	0.4	8.0	1.4	1.2	1.5	1.0	1.6	2.3	0.8	38.8	17.0	16.9
ACICU_02868		dolichyl-phosphate-mannose-protein mannosyltransferase			4.4	3.4	3.6	5.2	4.7	50.3	2.5	2.8	3.8	4.7	5.5	4.7	4.8	25.1	1.9	2.5	5.6	2.6	5.0	3.6	3.7	52.7	22.5	38.4
ACICU_02895		peptide synthetase			4.9	4.2	6.6	26.5	27.6	187.1	4.6	4.0	5.0	7.1	12.4	11.5	4.4	54.8	4.1	4.9	4.3	3.1	5.1	6.2	4.7	228.2	48.1	190.5
ACICU_02907	Y	diacylglycerol kinase	pmr related	mutation	13.0	10.9	15.7	54.2	52.7	219.4	7.8	10.4	10.6	20.5	26.6	26.3	25.1	119.4	7.5	14.1	11.9	13.2	16.7	13.2	19.4	209.0	548.7	204.1
ACICU_02938	Y	type I secretion protein	ACICU_02946 ISAba27 upstream in 588 isolates, genes between ACI(	insertion sequence	278.5	221.4	243.4	191.3	226.6	142.8	251.9	91.5	267.6	196.0	243.4	229.2	463.8	284.5	962.8	728.7	311.0	671.6	388.9	670.2	47.8	61.9	800.4	979.5
ACICU_02980		hypothetical protein	None ID	none identified	12.3	21.9	23.1	20.2	22.3	20.9	22.3	29.9	25.9	69.9	63.8	82.8	30.1	27.4	22.2	16.4	16.9	73.4	117.0	84.6	117.3	136.2	149.2	141.9
ACICU_03000		ammonium transporter	None ID	none identified	30.4	13.3	8.6	16.4	16.2	15.1	26.9	7.5	20.7	18.0	13.1	16.0	18.6	9.2	107.7	114.1	12.8	24.0	9.4	41.6	12.2	13.1	10.2	24.7
ACICU_03001		hypothetical protein	pmr related	mutation	3.9	2.5	2.9	8.1	13.8	61.3	2.4	1.6	2.0	4.8	6.9	6.6	3.8	29.1	1.1	4.9	1.9	1.9	4.5	6.0	4.6	69.2	18.2	57.7
ACICU_03002		pmrB histidine kinase			38.6	35.3	29.9	61.8	73.0	308.7	34.7	37.8	50.8	60.5	59.4	64.3	50.6	162.3	34.5	44.4	39.0	38.7	54.7	49.5	63.9	232.8	230.1	255.5
ACICU_03003		pmrA transcriptional regulator			10.8	15.6	14.5	33.6	34.2	187.3	11.4	20.4	11.7	16.1	25.4	25.5	16.8	76.7	10.4	16.8	12.6	23.0	22.4	16.4	23.1	145.1	121.1	176.8
ACICU_03004		pmrC lipid A phosphoethanolamine transferase			4.5	3.1	7.0	39.0	36.4	268.5	2.8	2.0	3.0	4.9	24.7	17.5	4.7	100.2	1.0	3.4	4.7	3.8	8.7	5.0	6.1	196.1	164.0	245.3
ACICU_03044		sodium:alanine symporter		none identified	20.0	9.1	12.6	10.4	11.0	12.3	8.5	24.9	20.6	36.7	41.8	40.2	15.6	15.3	30.4	13.3	24.4	64.6	20.9	54.4	33.9	82.3	81.4	65.2
ACICU_03076		oxidoreductase	ISAba1 in clade A and B	insertion sequence	22.4	27.1	27.6	18.2	17.9	29.3	28.4	35.9	53.9	85.4	62.7	93.1	92.9	37.8	39.4	42.3	25.5	111.1	225.5	105.0	226.3	196.9	171.5	118.2
ACICU_03111		peroxidase	None ID		239.2	591.4	518.1	171.4	142.7	121.4	453.5	96.7	232.0	463.4	494.2	916.5	961.9	492.1	250.7	366.8	174.2	612.2	1615.0	702.0	1191.8	811.9	639.3	475.3
ACICU_03285		flavohemoprotein	None ID	none identified	780.1	897.3	306.0	324.4	2068.7	1431.5	2091.3	1110.0	312.5	170.3	103.6	98.3	208.8	359.9	474.0	2155.2	500.4	70.8	57.5	148.1	164.0	145.5	256.3	167.1
ACICU_03286		transcriptional regulator			79.6	112.8	56.4	51.6	243.8	134.5	229.1	110.9	33.9	42.3	36.9	28.5	20.2	48.1	31.4	166.7	66.6	34.6	41.0	37.4	42.2	34.0	33.9	37.8
ACICU_03305		DEAD/DEAH box helicase	sequence divergence here, A&B same vs C&D same(9 mismatch, 1 ga	sequence variation	7325.5	6995.4	7520.2	7364.2	8703.4	4550.9	9789.3	2970.6	5137.9	5185.9	5449.7	4544.3	3628.5	3612.1	4883.6	4180.5	5319.0	7017.6	2245.9	7292.2	1163.1	1730.3	2236.4	1719.4
ACICU_03344		superoxide dismutase	None ID	none identified	101.4	167.1	117.3	73.1	64.4	71.4	136.6	66.2	130.2	231.3	258.1	299.0	240.7	176.4	192.0	159.7	93.0	373.3	787.5	315.6	779.4	301.2	327.9	341.1
ACICU_03382		hypothetical protein	None ID	none identified	16.4	31.4	28.2	22.5	17.8	30.5	29.7	18.8	22.2	78.6	72.5	96.1	40.3	39.4	24.7	33.3	32.5	129.5	134.3	130.9	215.0	142.1	155.0	91.3
ACICU_03394	Y	pilus assembly protein PilM	sequence divergence between clade D and others, but otherwise identic	sequence variation	224.0	80.3	82.0	304.2	270.2	347.0	146.1	245.8	186.6	158.2	93.2	85.8	98.3	126.3	102.4	104.7	167.3	35.9	26.9	49.9	44.2	67.7	66.0	57.2
ACICU_03422		sulfate permease	sequence divergence between A,C vs B, D	sequence variation	13.9	19.5	31.0	12.3	10.6	21.3	22.7	7.2	51.6	84.8	84.1	120.9	62.7	65.0	30.6	27.0	16.4	32.1	51.6	22.3	121.9	431.5	91.9	176.0
ACICU_03437		membrane protein	clade B ISAba1 in ACICU_03437	insertion sequence	82.9	66.3	45.6	69.4	65.6	74.2	50.3	59.4	96.8	48.6	50.6	49.1	23.4	16.0	11.3	13.7	21.2	54.7	50.0	74.2	79.2	87.2	103.4	113.3
ACICU_03447		hypothetical protein	None ID	none identified	59.7	127.1	152.2	30.7	35.4	38.9	197.6	53.9	271.0	227.9	219.2	328.4	471.9	214.3	175.8	235.1	110.1	250.2	713.9	368.4	894.6	738.5	257.1	209.9
ACICU_03477		4-aminobutyrate aminotransferase	mutation in ACICU_03477, in 667276	mutation	105.8	214.4	174.6	201.9	199.9	256.9	125.0	172.4	80.1	46.1	85.6	98.7	273.8	314.6	282.1	251.9	2404.9	280.9	302.1	278.8	278.1	210.0	128.7	114.0
ACICU_03479		gamma-aminobutyrate transporter			82.7	102.3	81.3	114.7	110.5	163.2	71.5	138.1	64.8	41.0	54.2	64.6	188.7	179.1	148.2	132.0	1368.2	141.9	165.2	160.7	167.7	164.9	73.6	70.8
ACICU_03576		ABC transporter substrate-binding protein	sequence divergence between D vs A-C, predicted to be anitgenic pepti	sequence variation	39.9	53.9	50.3	27.6	22.9	21.1	57.8	6.7	61.6	95.7	106.3	166.3	58.2	50.5	23.4	46.9	25.3	52.5	141.0	64.6	38.0	43.5	13.2	23.0
ACICU_03585		hypothetical protein	None ID	none identified	3850.7	953.8	847.3	2776.2	4034.9	3568.7	1931.2	2317.9	2087.4	1924.3	1127.3	1141.9	2108.4	1904.3	2302.0	3206.2	1911.8	590.3	398.8	977.0	1077.8	1398.9	1103.8	1651.7
ACICU_03609		aromatic amino acid transporter	PR ISAba125 in ACICU_03611	insertion sequence	9.9	19.9	13.4	12.4	9.4	11.4	6.7	11.7	4.7	8.1	207.4	209.5	15.6	14.4	11.8	7.5	8.5	8.3	7.7	8.8	9.5	8.7	70.6	57.5
ACICU_03610		fumarylacetoacetase			75.1	188.0	115.1	134.1	82.7	125.7	96.2	76.2	27.3	51.8	3255.8	2555.0	234.6	160.5	98.3	105.2	39.6	33.7	32.8	48.8	35.9	46.6	291.0	274.5
ACICU_03614		4-hydroxyphenylpyruvate dioxygenase			48.6	104.9	91.3	83.5	48.5	94.3	46.8	106.7	30.8	38.8	1576.1	1675.4	79.3	77.6	79.0	54.4	38.2	22.3	33.6	31.6	62.8	76.1	256.0	248.7
ACICU_03627	Y	membrane protein	None ID	none identified	232.5	454.6	578.2	133.2	116.3	74.1	425.9	50.5	175.1	594.5	538.6	516.6	817.3	759.8	255.0	434.7	211.4	1079.9	1885.4	667.8	1073.5	536.5	133.7	292.4
			sequence variation	11																								
			insertion sequence	21																								
			mutation	9																								
