# Supplementary material for: Transcriptome Remodeling of Acinetobacter baumannii during Infection and Treatment
Source: mBio. 2017 Mar 7;8(2):e02193-16. doi: 10.1128/mBio.02193-16 (PMC5340874; doi:10.1128/mBio.02193-16)
Supplement: TABLE S6 [file mbo001173221st6.pdf]

Table S6. *crp* and *osmC* expression values and predicted copy number of composite transposon

clade		A												B					C					D	
locus tag	predicted annotation	ABUH315100	ABUH315101	ABUH81366	ABUH81389	ABUH81452	ABUH28081	ABUH28092	ABUH28093	ABUH28099	ABUH475197	ABUH475239	ABUH475361	ABUH66241	ABUH66253	ABUH66268	ABUH66271	ABUH66276	ABUH41096	ABUH410103	ABUH410128	ABUH588656	ABUH588663	ABUH34813	ABUH34827
ACICU_01161*	crp transcriptional regulator	617.1	501.3	758.7	466.0	665.2	565.3	828.6	981.4	703.1	791.5	1112.4	1340.4	276.2	415.1	287.8	350.3	313.5	239.5	571.8	323.3	340.3	247.3	360.2	287.8
ACICU_01162	osmotically inducible protein C	420.4	401.6	539.1	351.8	352.8	471.7	576.3	757.2	606.5	594.5	542.8	657.3	203.7	219.7	200.6	248.7	185.3	161.1	332.8	229.3	119.9	171.6	207.5	169.1
**IS*Aba*1 status**		IS*Aba*1 in ACICU_01161 and IS*Aba*1 downstream of ACICU_01162												IS*Aba*1 in ACICU_01161										no IS*Aba*1	
**predicted number of crp and osmC transposon copies**		2	2	2	2	2	3	3	3	3	3	3	3	0	0	0	0	0	0	0	0	0	0	0	0

*mean normalized read counts from DESeq2
